# Effect of dietary restriction and subsequent re-alimentation on the transcriptional profile of bovine ruminal epithelium

**DOI:** 10.1371/journal.pone.0177852

**Published:** 2017-05-17

**Authors:** Kate Keogh, Sinead M. Waters, Paul Cormican, Alan K. Kelly, Emma O’Shea, David A. Kenny

**Affiliations:** 1 Animal and Bioscience Research Department, Animal and Grassland Research and Innovation Centre, Teagasc, Grange, Dunsany, Co. Meath, Ireland; 2 School of Agriculture and Food Science, University College Dublin, Belfield, Dublin, Ireland; Northwest A&F University, CHINA

## Abstract

Compensatory growth (CG) is utilised worldwide in beef production systems as a management approach to reduce feed costs. However the underlying biology regulating the expression of CG remains to be fully elucidated. The objective of this study was to examine the effect of dietary restriction and subsequent re-alimentation induced CG on the global gene expression profile of ruminal epithelial papillae. Holstein Friesian bulls (n = 60) were assigned to one of two groups: restricted feed allowance (RES; n = 30) for 125 days (Period 1) followed by *ad libitum* access to feed for 55 days (Period 2) or (ii) *ad libitum* access to feed throughout (ADLIB; n = 30). At the end of each period, 15 animals from each treatment were slaughtered and rumen papillae harvested. mRNA was isolated from all papillae samples collected. cDNA libraries were then prepared and sequenced. Resultant reads were subsequently analysed bioinformatically and differentially expressed genes (DEGs) are defined as having a Benjamini-Hochberg P value of <0.05. During re-alimentation in Period 2, RES animals displayed CG, growing at 1.8 times the rate of their ADLIB contemporary animals in Period 2 (P < 0.001). At the end of Period 1, 64 DEGs were identified between RES and ADLIB, with only one DEG identified at the end of Period 2. When analysed within RES treatment (RES, Period 2 v Period 1), 411 DEGs were evident. Genes identified as differentially expressed in response to both dietary restriction and subsequent CG included those involved in processes such as cellular interactions and transport, protein folding and gene expression, as well as immune response. This study provides an insight into the molecular mechanisms underlying the expression of CG in rumen papillae of cattle; however the results suggest that the role of the ruminal epithelium in supporting overall animal CG may have declined by day 55 of re-alimentation.

## Introduction

In beef cattle production, profitability is driven by the efficient conversion of feed to carcass growth. Given that feed inputs typically account for up to 75% of the variable costs [1] any improvement in lifetime nutrient utilization will enhance economic efficiency of beef production systems. Moreover, enhanced feed efficiency can lead not only to improved profitability but has also been shown to result in a reduction in ruminal methane emissions [2, 3] therefore reducing the carbon footprint of beef production. Compensatory growth (CG) is an accelerated growth rate typically observed following a period of under-nutrition to facilitate an animal in reaching its genetically pre-determined growth potential [4]. The exploitation of the CG phenomenon is one management strategy implemented by producers to reduce the overwintering feed costs of cattle production [5, 6] and is utilised worldwide [7–11]. The well documented phenotypic variation amongst similarly managed cattle in their CG response [11–14] suggests that this process is genetically controlled. However although widely studied, the underlying molecular mechanisms controlling the expression of CG, remain to be determined.

The rumen accounts for 80% of the entire ruminant foregut and following ruminal digestion, volatile fatty acids (VFAs) and microbial protein are the primary end-products [15]. The VFAs provide approximately 80% of the metabolizable energy requirements of the animal [16], whilst microbial protein typically provides 50–80% of the crude protein that reaches the small intestine [17]. Digestive tract tissues in ruminants have been shown to be responsive to changes in dietary protein and energy intake [18–21] as well as to nutrient restriction [18, 22]. Indeed, components of the gastrointestinal tract, including the rumen, have repeatedly been shown to undergo CG ahead of other tissues and organs [7, 8, 11]. CG profiles for various organs and tissues suggest that ruminal tissue receives priority for nutrients ahead of tissues less associated with digestion and metabolism [7, 8, 11]. Indeed, we previously reported this in the animals used in the current study [11]. Moreover, a study by Sun et al. [23] showed that rumen papillae height, width and surface area were all lower in goats that had undergone a 6 week period of dietary restriction. However following a period of re-alimentation, rumen epithelial tissues were not found to be morphologically different to that of unrestricted animals [23].

Therefore, the objective of the current study was to quantify changes in the rumen papillae transcriptome of beef cattle in response to nutrient restriction and subsequent CG, with a view to examining the contribution of this tissue to the overall biochemical regulation of CG in cattle. Ultimately the data generated in this study will be combined with the outcomes of other recently published studies from our laboratory, across a number of metabolically important tissues [24–26], to assist the identification of key candidate genes underpinning feed efficient growth in beef cattle that could be further exploited within the context of genomically assisted breeding programs for beef cattle.

## Materials and methods

All procedures involving animals were approved for the use of live animals in experiments by the University College Dublin, Animal Research Ethics Committee and were licensed by the Irish Department of Health and Children, in accordance with the Cruelty to Animals Act (Ireland 1897) and European Community Directive 86/609/EC.

### Animal model and management

This experiment was conducted as part of a research programme designed to examine the physiological and molecular control of CG in growing beef cattle [11, 12]. Purebred Holstein Friesian bulls (n = 60) were managed on the same commercial farm in Co. Offaly, Ireland, prior to being transferred to Teagasc Grange Beef Research Centre, Dunsany, Co. Meath, Ireland. In order to acclimatise the animals to their environment and reduce any latent influence of their previous environment, all animals were subjected to a 3 month common feeding period consisting of grass silage offered *ad libitum* plus 2 kg of concentrate per head per day. Animals (mean live-weight 370 ± 35 kg; mean age 479 ± 15 d) were blocked on the basis of live-weight and age and assigned within block to one of two dietary regimens: (i) restricted feed allowance for 125 days (RES; n = 30) followed by *ad libitum* access to feed for a further 55 days (RES; n = 15) or (ii) *ad libitum* access to feed throughout the trial (ADLIB; n = 30). The first 125 days of the trial were denoted as Period 1 and the subsequent 55 days, Period 2. Animals in the control group (ADLIB) were offered a 70:30 concentrate: forage (grass silage) diet *ad libitum* throughout the trial. The remaining 30 bulls (RES) were offered a restricted quantity of the same diet. Target growth rates for RES and ADLIB were 0.6 kg day^-1^ and in excess of 1.5 kg day^-1^ during Period 1, respectively. The concentrate ration consisted of rolled barley (72.5%), soyabean meal (22.5%), molasses (3%) and mineral supplement (2%). Chemical composition is described in more detail by Keogh et al. [11]. Diets were fed individually, with the proportion of feed required, based on each animal’s own individual bodyweight. Animals were weighed on two consecutive days at the start of the study, at the end of Period 1 and again at the end of Period 2. Additionally, throughout the study, animals were weighed every two weeks during Period 1 and every week during Period 2. Weighing was conducted at the same time each morning, before fresh feed was offered. During the study animals were managed under strict animal welfare guidelines and were under the daily care of trained herdsmen. The health and welfare of the animals were also routinely monitored by the designated veterinary surgeon who visited the facility on a daily basis.

Following completion of Period 1, 15 animals from each treatment (RES and ADLIB) were slaughtered. Prior to the commencement of Period 2, RES were allowed a 15 day transition period in order to build up to *ad libitum* feed intake. This was to obviate potential metabolic disorders such as ruminal acidosis. All remaining bulls (n = 30) were then offered the control diet *ad libitum* for a further 40 days before slaughter.

### Tissue sample collection

All animals were humanely slaughtered via captive bolt stunning followed by exsanguination in an EU licensed abattoir (Euro Farm Foods Ltd, Cooksgrove, Duleek, Co. Meath, Ireland) and all tissue samples were harvested post slaughter. Slaughter order at the abattoir was randomized to account for any potential confounding effects on treatment outcomes. The abattoir was located within 30 mins drive of the research station. The duration from slaughter to evisceration was no more than 30 mins and was consistent for all animals, irrespective of treatment. Tissue samples were excised post-mortem from the ventral sac of the rumen within 40 min of slaughter [27]. All instruments used for tissue collection were sterilized and treated with RNase Zap prior to use (Ambion, Applera Ireland, Dublin, Ireland). Rumen papillae were harvested directly using scissors. Samples were washed thoroughly with sterile, RNase free, phosphate buffered saline (PBS) and subsequently snap frozen in liquid nitrogen before being stored at -80°C.

### RNA extraction and purification

Total RNA was isolated from approximately 100 mg of frozen rumen papillae tissue using TRIzol reagent and chloroform (Sigma-Aldrich Ireland, Dublin, Ireland). Tissue samples were homogenised using a rotor-stator tissue lyser (Qiagen, UK), following which the RNA was precipitated using isopropanol. Samples were then treated with an RNeasy Plus Mini Kit (Qiagen, UK), according to the manufacturer’s instructions in order to remove any contaminating genomic DNA. The quantity of the RNA isolated was determined by measuring the absorbance at 260 nm using a Nanodrop spectrophotometer ND-1000 (Nano Drop Technologies, LLC, Wilmington, DE, USA). RNA quality was assessed on the Agilent Bioanalyser 2100 using the RNA 6000 Nano Lab Chip kit (Agilent Technologies Ireland Ltd., Dublin, Ireland). RNA quality was also verified by ensuring all RNA samples had an absorbance (A260/280) ratio of between 1.8 and 2. RNA samples with 28S/18S ratios ranging from 1.8 to 2.0 and RINs (RNA integrity number) of between 8 and 10 were deemed high quality. High quality RNA samples were selected from 10 representative animals within each treatment from each period.

### cDNA library preparation and sequencing

cDNA libraries were prepared from high quality RNA (3 μg per sample) using an Illumina TruSeq RNA sample prep kit following the manufacturer’s instruction (Illumina, San Diego, CA, USA). Briefly, mRNA was isolated from total RNA and strands subsequently fragmented. First strand cDNA was synthesised using SuperScript^®^ II Reverse Transcriptase (Applied Biosystems Ltd., Life Technologies, Warrington, UK), second strand synthesis was subsequently performed using components supplied in the Illumina TruSeq RNA sample prep kit. Indexing adaptors were ligated to the cDNA which was then enriched through PCR. Final individual cDNA libraries were validated on the Agilent Bioanaylser 2100 using the DNA 1000 Nano Lab Chip kit, ensuring that library fragment size was ~260 bp and library concentration was >30 ng/μl. After quality control procedures, individual RNA-seq libraries were pooled based on their respective sample-specific-6bp adaptors and sequenced at 100 bp/sequence on an Illumina HiSeq 2000 generating single-end reads.

### Read alignment and abundance calculation

Preliminary quality control analysis was carried out using FASTQC software (version 0.10.0). FASTX-Toolkit (v0.0.13) was then used to trim 3’ adaptor sequences. Trimmed reads were then subsequently aligned to the UMD3.1 *Bos Taurus* genome assembly using Tophat (v2.0.9) and Bowtie2 ultra-fast short read alignment software (v2.1.0). The software package HTSeq (v0.5.4p5) (http://pypi.python.org/pypi/HTSeq) was employed to calculate the abundance of mRNAs for all annotated genes from the ENSEMBL v74 annotation of the bovine genome. The number of read counts mapping to each annotated gene from HTSeq was then collated into a single file and subsequently used to identify differentially expressed genes (DEGs).

### Identification of DEGs

The R (v2.14.1) based Bioconductor package, EdgeR (v3.4.1), which uses a negative binomial distribution model to account for both biological and technical variability, was employed to identify statistically significant DEGs. Genes with low read counts across all libraries were excluded from subsequent analysis. The analysis was undertaken using moderated tagwise dispersions. DEGs were defined as having a Benjamini-Hochberg corrected P value of < 0.05 and a false discovery rate (FDR) of <0.1%. Data analysis was undertaken to determine genes differentially expressed in RES animals relative to ADLIB animals at each time-point (periods 1 and 2). Additionally, data pertaining to both RES and ADLIB groups at each time-point were analysed within treatment group; DEGs were identified in RES Period 2 relative to RES Period 1 and ADLIB Period 2 relative to ADLIB Period 1.

### Pathway analysis

Pathway and functional analysis of DEGs was undertaken using Ingenuity Pathway Analysis (IPA) (v. 8.8, Ingenuity Systems, Mountain View, CA; http://www.ingenuity.com), a web-based software application that enables identification of over-represented biological mechanisms, pathways and functions most relevant to experimental datasets or genes of interest [28–30]. IPA analysis was used to identify biological functions, canonical pathways, networks and upstream regulators involved in the response to nutrient restriction and subsequent re-alimentation.

## Results

### Animal performance

The animal performance data pertaining to samples utilised in this study are presented and discussed in detail by Keogh et al. [11]. Briefly, following a period of 125 days of dietary restriction, there was a 161 kg difference in bodyweight between RES (mean (SEM); 442 (6.67) kg) and ADLIB (603 (7.21) kg) animals (P < 0.0001). After the subsequent re-alimentation period (55 days) this was reduced to 86 kg (RES: 594 (9.44) kg; ADLIB: 678 (9.87) kg) (P < 0.0001). Average daily gain (ADG) for Period 1 was 0.6 (0.05) kg/d for RES animals and 1.9 (0.05) kg/d for ADLIB animals (P < 0.0001). During re-alimentation (Period 2) an ADG of 2.5 (0.06) kg/d and 1.4 (0.07) kg/d was observed for RES and ADLIB groups, respectively (P< 0.001). Feed conversion ratio (feed efficiency index) was better in RES animals during re-alimentation induced CG in Period 2 compared to RES Period 1 and ADLIB animals across both periods, (Period 1: RES: 9.5 (0.45); ADLIB: 6.71 (0.48); Period 2: RES: 4.87 (0.63); ADLIB: 9.98 (0.69); P < 0.0001). Reticulo-rumen weights (empty of digesta) were lighter in RES animals (0.169 (0.0006) kg/kg BW) compared to ADLIB (0.0195 (0.0007) kg/kg BW) at the end of Period 1 (P < 0.05). However, no difference in reticulo-rumen weight was apparent at the end of Period 2 (RES: 0.219 (0.0006) kg/kg BW; ADLIB: 0.0206 (0.0007) kg/kg BW; P > 0.05) [11].

### Read mapping and differential gene expression

Approximately 86% of sequencing reads (after trimming) were aligned to the bovine genome and 73% of those that aligned were mapped to the gene space. The bovine reference genome (UMD3.1) contains 26,740 gene transcripts. At the end of dietary restriction in Period 1, the number of genes that had mapped reads was 12,634, whereas following 55 days of re-alimentation in Period 2, 12,711 genes had reads mapping to them. Using the bioconductor package EdgeR, 64 genes were identified as differentially expressed between RES and ADLIB animals at the end of Period 1. These were manifested as up-regulation of 40 and down-regulation of 24 genes in RES animals compared to ADLIB treatment. Further details of these genes are provided in S1 Table. Following 55 days of subsequent *ad libitum* feeding only one gene was differentially expressed between RES and ADLIB. *BNBD10*, a beta defensin gene, was down-regulated in RES animals compared to ADLIB animals at the end of Period 2. Additionally, when the data were examined within the RES treatment, 411 genes were identified as differentially expressed between periods 1 and 2. From this latter analysis 226 genes were down-regulated and 185 up-regulated in RES during Period 2 compared with Period 1. Further details of these genes are provided in S2 Table. Data pertaining to the ADLIB group across time resulted in differential expression of 5 genes, these included *MAOB* and *DNAJC6* which both had greater expression at the end of Period 2 compared to Period 1 and *NQO2*, *ADAMTSL3* and *CCL22*, which had lower expression at the end of Period 2 compared to Period 1. These RNA-seq data have been deposited in the NCBI’s Gene Expression Omnibus [31] and are accessible through GEO Series accession number GSE89162.

### Pathway analysis

**Fig 1:**
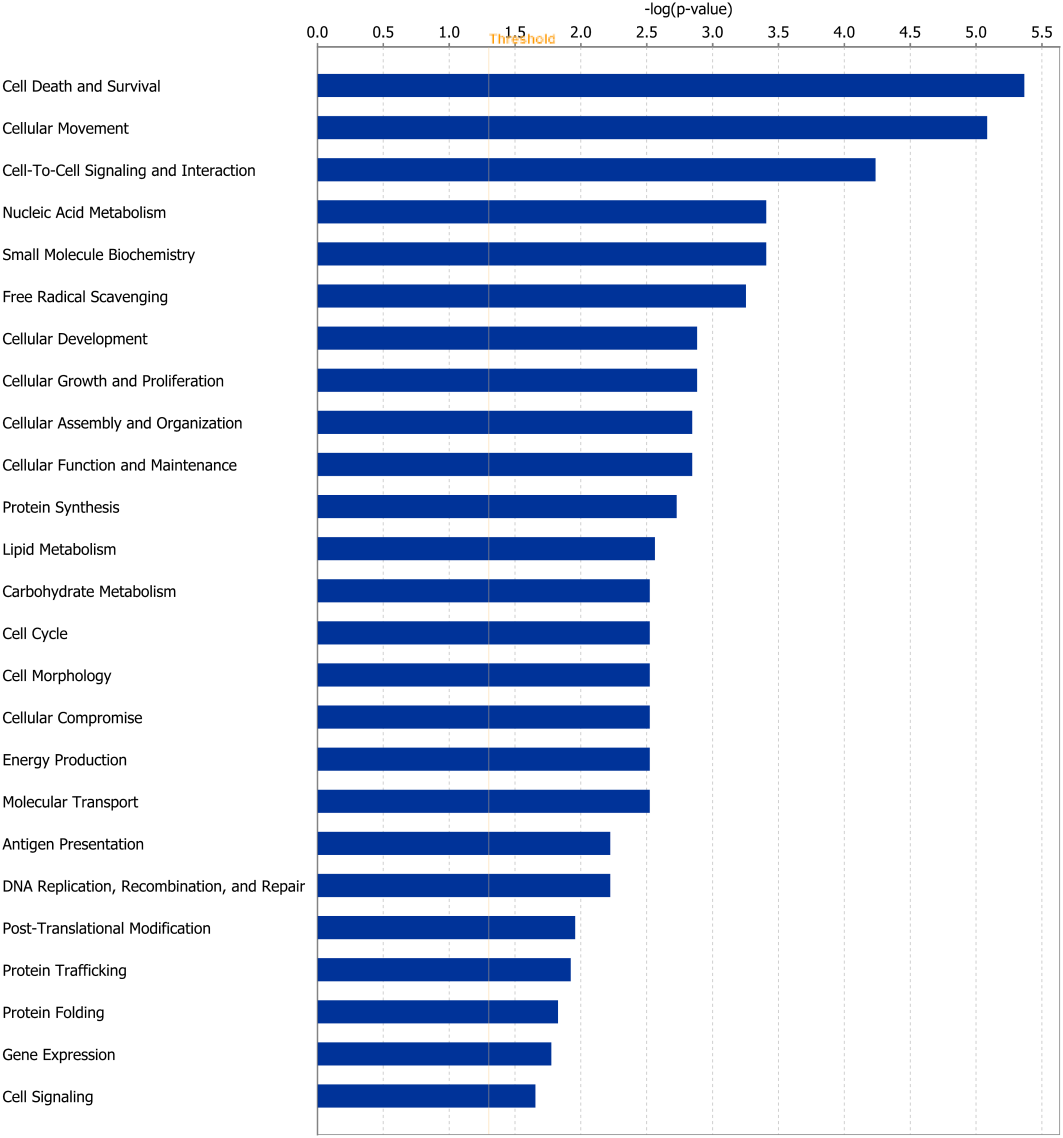
Classification of differentially expressed genes according to molecular and cellular function, most significantly affected by restricted feeding in rumen papillae at the end of Period 1. The bars indicate the likelihood [-log(P value)] that the specific molecular and cellular function was affected by restricted feeding compared with other functions represented in the list of differentially expressed genes.

**Fig 2:**
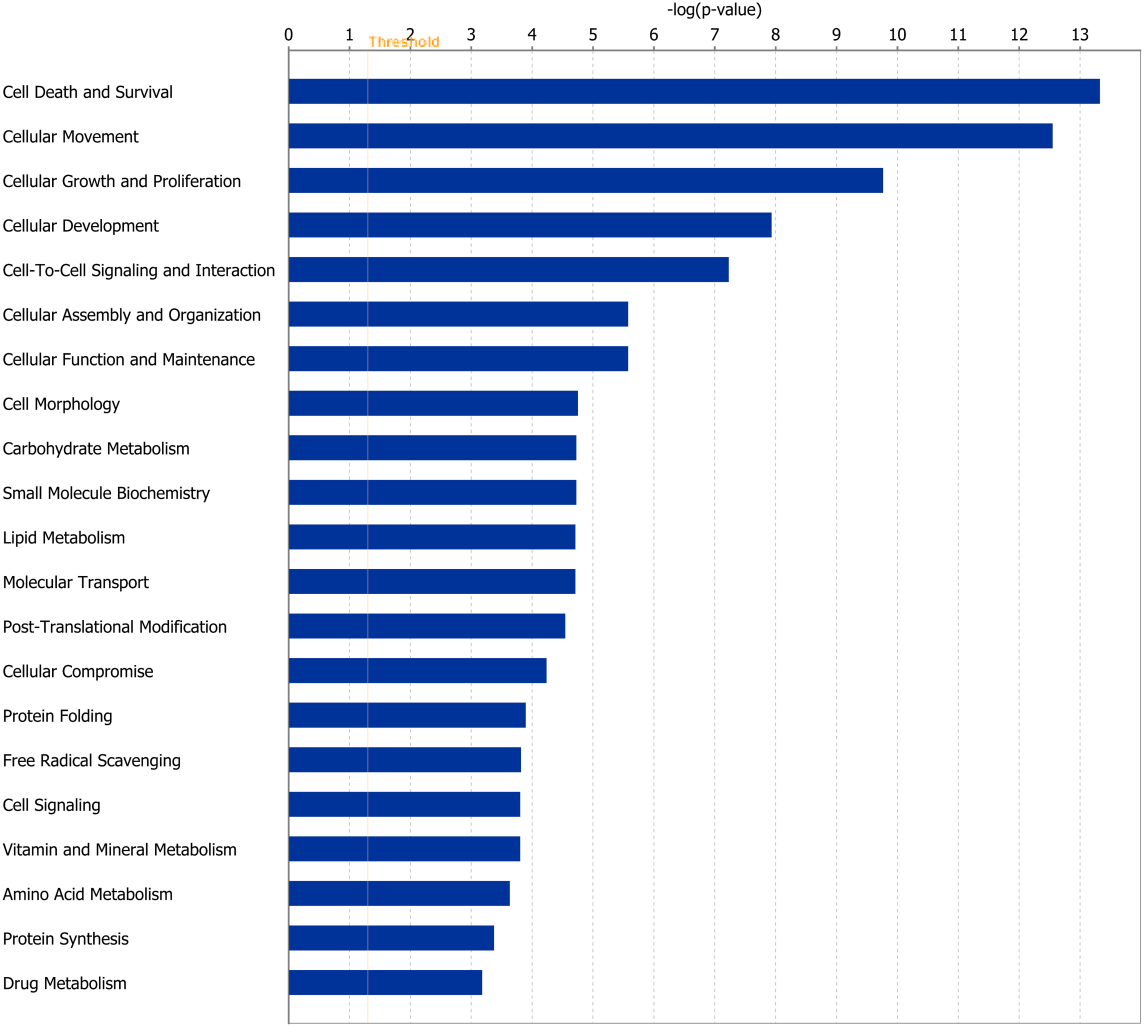
Classification of differentially expressed genes according to molecular and cellular function, most significantly affected by re-alimentation and compensatory growth in rumen papillae. The bars indicate the likelihood [-log(P value)] that the specific molecular and cellular function was affected by re-alimentation induced CG compared with other functions represented in the list of differentially expressed genes.

DEGs were analysed and separated according to their biological functions using IPA software. At the end of Period 1, genes involved in processes including cellular signalling and interaction, protein synthesis and gene expression were differentially expressed. The direction of fold change for DEGs within these processes indicated an overall down-regulation of these cellular functions in rumen papillae in response to dietary restriction. During CG of RES papillae in Period 2 genes coding for proteins involved in cellular survival/organisation and protein folding were differentially expressed compared with dietary restriction in Period 1. The direction of fold change of these genes suggested an up-regulation of these processes during CG. Further details of the genes involved in these processes are outlined in Tables 1, 2 and 3 (Table 1: gene expression and protein folding; Table 2: cellular interactions and organisation; Table 3: immune response). Details of functional processes affected by dietary restriction and subsequent re-alimentation induced CG are presented in Figs 1 and 2 respectively.

**Table 1:** Genes involved in gene expression and protein folding functions found to be differentially expressed in ruminal papillae following: A period of (i) dietary restriction and (ii) re-alimentation induced compensatory growth.

Gene ID	Gene name	Fold change^1^
**Dietary restriction**		
*CRYAB*	Crystallin, alpha B	-1.708
*HSPB8*	Heat shock 22kDa protein 8	-1.466
*HSPH1*	Heat shock 105kDa/110kDa protein 1	-1.628
*SATB1*	SATB homeobox 1	2.354
*ZC3H12A*	Zinc finger CCCH-type containing 12A	1.748
**Compensatory growth**		
*AHSA1*	AHA1, activator of heat shock 90kDa protein ATPase homolog 1 (yeast)	1.491
*DNAJB4*	DnaJ (Hsp40) homolog, subfamily B, member 4	1.304
*HSPA8*	Heat shock 70kDa protein 8	1.543
*HSPB8*	Heat shock 22kDa protein 8	1.739
*HSPD1*	Heat shock 60kDa protein 1 (chaperonin)	1.306
*MDN1*	MDN1, midasin homolog (yeast)	1.386
*CCT2*	Chaperonin containing TCP1, subunit 2 (beta)	1.262
*HSP90AA1*	Heat shock protein 90kDa alpha (cytosolic), class A member 1	1.697
*HSP90AB1*	Heat shock protein 90kDa alpha (cytosolic), class B member 1	1.284
*HSPE1*	Heat shock 10kDa protein 1	1.375
*PPID*	Peptidylprolyl isomerase D	1.392
*STIP1*	Stress-induced phosphoprotein 1	1.436
*EMG1*	EMG1 N1-specific pseudouridine methyltransferase	1.267
*FOXN1*	Forkhead box N1	2.581
*FOXP4*	Forkhead box P4	1.403
*INTS3*	Integrator complex subunit 3	1.28
*KHDRBS3*	KH domain containing, RNA binding, signal transduction associated 3	1.488
*PRCC*	Papillary renal cell carcinoma (translocation-associated)	1.253
*EIF4G2*	Eukaryotic translation initiation factor 4 gamma, 2	1.250
*EIF4G3*,	Eukaryotic translation initiation factor 4 gamma, 3	1.251
*ELL2*	Elongation factor, RNA polymerase II, 2	1.423
*HIST1H2AC*	Histone cluster 1, H2ac	2.538
*HIST1H2BD*	Histone cluster 1, H2bd	1.595
*HIST1H2BN*	Histone cluster 1, H2bn	1.618
*HISTH2BO*	Histone cluster 1, H2bo	1.331
*HIST2H4A*	Histone cluster 2, H4a	1.634
*KAT2A*	K(lysine) acetyltransferase 2A	1.250

^1^ Fold changes are as follows: (i) dietary restriction: up or down in restricted fed animals compared with *ad libitum* control animals during dietary restriction at the end of Period 1; (ii) compensatory growth: up or down in restricted Period 2 animals compared to restricted Period 1 animals during compensatory growth.

**Table 2:** Genes involved in cellular interactions and organisation differentially expressed in rumen papillae following a period of (i) dietary restriction and (ii) re-alimentation induced compensatory growth.

Gene ID	Gene name	Fold change^1^
**Dietary restriction**		
*CDH2*	Cadherin 2, type 1, N-cadherin (neuronal)	-2.895
*DSG1*	Desmoglein 1	-4.632
**Compensatory growth**		
*ANTXR1*	Anthrax toxin receptor 1	1.347
*CEP97*	Centrosomal protein 97kDa	1.461
*FAT4*	FAT atypical cadherin 4	1.609
*PCDH12*	Protocadherin 12	1.715
*PCDH7*	Protocadherin 7	1.666
*ITGA8*	Integrin, alpha 8	1.746
*NRG1*	Neuregulin 1	1.512
*RELN*	Reelin	1.647
*SMAGP*	Small cell adhesion glycoprotein	1.303
*THBS4*	Thrombospondin 4	1.818
*SLC1A5*	Solute carrier family 1 (neutral amino acid transporter), member 5	1.342
*SLC22A17*	Solute carrier family 22, member 17	2.296
*SLC25A15*	Solute carrier family 25 (mitochondrial carrier; ornithine transporter) member 15	1.276
*SLC25A26*	Solute carrier family 25 (S-adenosylmethionine carrier), member 26	1.251
*SLC30A6*	Solute carrier family 30 (zinc transporter), member 6	1.295
*SLC4A7*	Solute carrier family 4, sodium bicarbonate cotransporter, member 7	1.397
*SLC6A9*	Solute carrier family 6 (neurotransmitter transporter, glycine), member 9	1.349
*SLC9A1*	Solute carrier family 9, subfamily A (NHE1, cation proton antiporter 1), member 1	1.318
*CACNA1G*	Calcium channel, voltage-dependent, T type, alpha 1G subunit	1.975
*KCNC4*	Potassium channel, voltage gated Shaw related subfamily C, member 4	1.492

^1^ Fold changes are as follows: (i) dietary restriction: up or down in restricted fed animals compared with *ad libitum* control animals during dietary restriction at the end of Period 1; (ii) compensatory growth: up or down in restricted Period 2 animals compared to restricted Period 1 animals during compensatory growth.

**Table 3:** Genes involved in immune response differentially expressed in rumen papillae following a period of (i) dietary restriction and (ii) re-alimentation induced compensatory growth.

Gene ID	Gene name	Fold change^1^
**Dietary restriction**		
*IL17A*	Interleukin 17A	3.707
*LBP*	Lipopolysaccharide binding protein	1.74
**Compensatory growth**		
*BDKRB1*	Bradykinin receptor B1	-1.971
*CHI3L1*	Chitinase 3-like 1 (cartilage glycoprotein-39)	-3.03
*HPGD*	Hydroxyprostaglandin dehydrogenase 15-(NAD)	-1.674
*LTA4H*	Leukotriene A4 hydrolase	-1.276
*C5AR2*	Complement component 5a receptor 2	-1.605
*CD59*	CD59 molecule, complement regulatory protein	-1.458
*CCL19*	Chemokine (C-C motif) ligand 19	-4.237
*CCL20*	Chemokine (C-C motif) ligand 20	-2.965
*CXCL12*	Chemokine (C-X-C motif) ligand 12	-2.189
*CXCL17*	Chemokine (C-X-C motif) ligand 17	-2.691
*CXCL2*	Chemokine (C-X-C motif) ligand 2	-2.957
*CXCR4*	Chemokine (C-X-C motif) receptor 4	-1.646
*LYZ*	Lysozyme	-3.144
*SGSH*	N-sulfoglucosamine sulfohydrolase	-1.261
*CYBA*	Cytochrome b-245, alpha polypeptide	-1.418

^1^ Fold changes are as follows: (i) dietary restriction: up or down in restricted fed animals compared with *ad libitum* control animals during dietary restriction at the end of Period 1; (ii) compensatory growth: up or down in restricted Period 2 animals compared to restricted Period 1 animals during compensatory growth.

**Fig 3:**
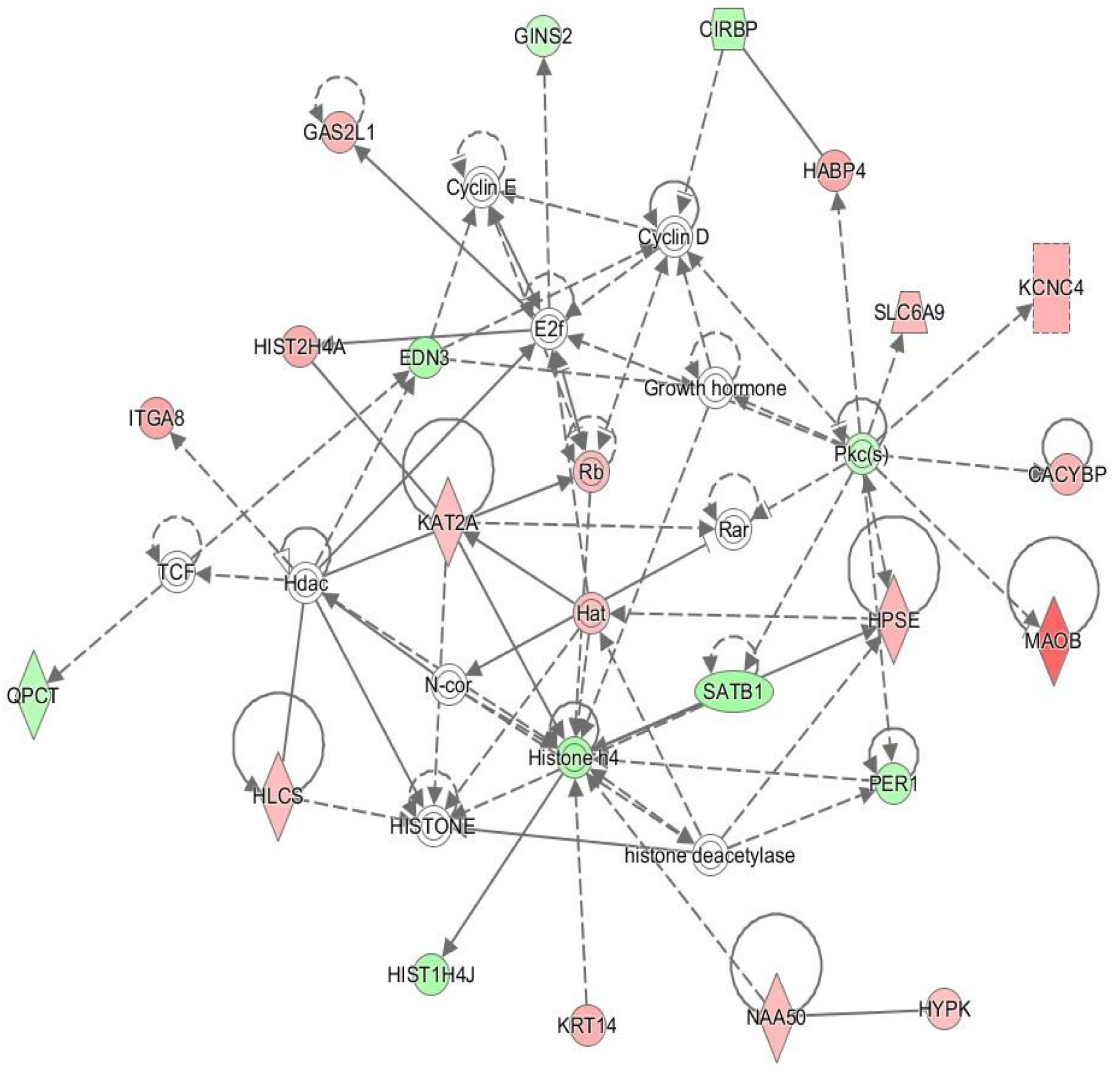
Metabolism and cellular assembly/organisation network in rumen papillae following re-alimentation induced compensatory growth (Network 6: Carbohydrate metabolism, small molecule biochemistry and cellular assembly and organisation). The network is displayed graphically as nodes (genes). The node colour intensity indicates the expression of genes; with red representing up-regulation and green, down-regulation in animals following a period of re-alimentation induced compensatory growth relative to following a period of dietary restriction.

Using IPA software, a total of five networks were identified for DEGs at the end of Period 1 (S3 Table), with 25 networks identified in rumen papillae of animals undergoing CG (RES Period 2 v RES Period 1; S4 Table). Network 6 was of particular interest in rumen papillae undergoing CG. This network consisted of genes involved in carbohydrate metabolism, small molecule biochemistry and cellular assembly and organisation and details are presented in Fig 3.

## Discussion

The CG phenomenon, typically expressed upon re-alimentation following a prior period of dietary restriction has been associated with improved feed efficiency in a number of cattle studies [4, 8, 32] including for the animals employed in the current study [11]. Moreover, Sainz et al. [14] and Keogh et al. [11] both reported greater feed intake in compensating animals following re-alimentation. Greater feed intake combined with greater animal ADG and the rapid CG of the rumen suggest changes in the activity of the rumen or potentially differences in digestibility capacity or epithelial morphology within the rumen during CG may play a role in accelerated growth. Results from our own study are consistent with previous studies, showing compensating animals consumed a greater amount of feed per unit of body weight when compared to their *ad libitum* fed counterparts during Period 2 [11]. Furthermore, increases in digestibility have also been reported in cattle undergoing CG [33]. Indeed, Sun et al. [23] observed differences in rumen papillae height, width and surface area in goats following a 48 day period of dietary restriction compared to those that had not been diet restricted. However, following a subsequent period of re-alimentation induced CG, lasting 62 days there were no longer any detectable differences in rumen epithelium morphology between animals that had undergone dietary restriction and subsequent CG compared with their unrestricted counterparts [23]. Moreover, the rumen has repeatedly been shown to be one of the most responsive organs to both dietary restriction and also subsequent CG, as evidenced by both our own work [11] as well as by that of others [7, 8]. This may be due to the high metabolic rate associated with this organ, with a reduction in rumen size following a period of dietary restriction allowing for a reduction in associated basal metabolic energy requirements of the organ [8, 11, 34]. Indeed, a lowered basal metabolic rate is thought to sustain through to at least the early stages of re-alimentation and contribute to CG by allowing more energy to be partitioned towards growth as opposed to maintenance requirements [11, 34]. However, although this tissue is clearly affected by both dietary restriction and CG, knowledge of the underlying biology regulating the expression of CG in rumen epithelial or indeed any tissue of the gastrointestinal tract is still lacking. Therefore, the objective of this study was to quantify and characterize by gene function, the transcriptional changes in rumen papillae of beef cattle in response to both nutrient restriction and subsequent CG and also to determine the contribution of these changes to overall animal CG. This was achieved through an examination of DEGs in rumen papillae following a period of dietary restriction and also a period of subsequent re-alimentation compared to rumen papillae of animals that were fed continuously. Additionally, sequencing data were analysed within treatment group to further assess the effect of CG on transcriptional changes within rumen papillae. The large difference in DEGs between RES and ADLIB groups when analysed within treatment across time (RES: 411 DEGs, ADLIB: 5 DEGs) suggests that the RES within treatment group analysis is reflective of CG and not of normal growth as described in the ADLIB DEG profile. A greater knowledge of molecular changes occurring during CG of highly metabolically important organs such as the rumen may facilitate more accurate identification of animals with improved CG potential and thus the possible incorporation of this economically important information into genomically assisted cattle breeding programs.

### Gene transcription and protein folding

A reduction in feed intake is typically paralleled by a reduction in growth and overall cellular functions. Indeed, this was apparent in the papillae of RES animals following a period of dietary restriction, where genes involved in gene transcription and protein folding tended to be down-regulated compared with ADLIB animals. Specifically these DEGs were manifested as down-regulation of genes coding for proteins involved in chaperone functionality including *CRYAB*, *HSPB8* and *HSPH1*. The CRYAB protein displays chaperone-like activity and functions in preventing aggregation of various proteins under a wide range of conditions [35]. We also found this gene to be down-regulated in the skeletal muscle tissue of these same cattle during dietary restriction [25]. Both *HSPB8* and *HSPH1* code for heat shock proteins which also function in the prevention of aggregation of denatured proteins in cells [36]. *HSPH1* was also found to be down-regulated in skeletal muscle of cattle following a period of dietary restriction [25]. Furthermore, up-regulation of both *SATB1* and *ZC3H12A* was also apparent in the rumen papillae of RES animals at the same time-point. Both of these genes code for proteins involved in repressing transcription, thereby causing down-regulation of gene expression processes [37, 38]. *SATB1* encodes a matrix protein, which functions to recruit chromatic remodelling factors in order to regulate chromatin structure and gene expression and ultimately functions in transcriptional repression and gene silencing [39]. Consistent with this *ZC3H12A* displays RNase activity and functions in selectively degrading specific target mRNA species [40]. We also found *ZC3H12A* to be up-regulated in hepatic tissue of these cattle following a period of dietary restriction [26]. Down-regulation of genes involved in these processes following a period of dietary restriction may be reflective of a reduced requirement for nutrient processing and metabolism, coinciding with lower animal ADG and weight of the rumen complex, at the end of Period 1 [11]. Moreover, following a 125 day period of dietary restriction, when compared with a reference slaughter group at the start of dietary restriction, the proportional weight of the rumen in feed restricted animals was found to be lower [11], further evidencing a reduced metabolic requirement of this organ in response to dietary restriction. Lower expression of genes involved in cellular metabolism, following dietary restriction was also apparent in liver tissue of the same animals used in the current study [26].

While the aforementioned biological processes were down-regulated during dietary restriction, upon re-alimentation up-regulation of these functions was apparent which coincided with greater rumen and overall body growth rates [11]. Up-regulation of these processes during re-alimentation also coincided with a greater capacity for growth and requirement for cellular metabolism in the rumen tissue as well as in other organs within the body. For example, increased expression of genes associated with metabolism during CG was previously described in the hepatic tissue of the animals used in the current study [26]. Indeed greater transcription of genes coding for proteins involved in gene expression was evident in papillae of animals displaying CG. Greater expression of these genes may be necessary in order to allow for increased production of proteins to accommodate the increased nutrient availability and metabolic demands of digestion, absorption and ultimately tissue growth. Genes involved in protein folding included those coding for chaperone proteins: *AHSA1*; *DNAJB4*; *HSPA8*; *HSPB8*; *HSPD1*; *MDN1*; as well as those requiring input of ATP: *CCT2*; *HSP90AA1*; *HSP90AB1*; *HSPE1*. These genes have also been shown to display greater expression during CG in other tissues and organs, including skeletal muscle (*AHSA1*, *DNAJB4*, *HSPA8*, *HSPB8* and *HSPD1*; [25]; and liver (*HSPA8*, *HSPB8* and *HSPD1*; [10]). Moreover, *HSPA8*, *HSPB8* and *HSPD1* were also up-regulated in the liver of feed efficient cattle [41]. These genes may be important in relation to improved feed efficiency consistent with that observed for cattle undergoing CG including those in the current study [4, 11]. Greater expression of *CCT2* and *HSPE1* was also reported in skeletal muscle of our animals while undergoing CG [25]. Furthermore, up-regulation of *HSP90AA1* and *HSP90AB1* was also apparent in both hepatic and skeletal muscle tissues of cattle undergoing CG [10, 25]. Greater expression of *PPID*, a protein that functions in accelerating protein folding [42] as well as *STIP1* which regulates both the conformation and ATPase cycles of HSP70 and HSP90 molecular chaperones [43] was also apparent in rumen papillae of cattle undergoing CG in the current study. Both of these genes were also up-regulated in skeletal muscle tissue of cattle undergoing CG [25]. Overall these results suggest that increased cellular protein folding activity is required within the rumen papillae as part of the adaption to an increased dietary intake and is consistent with the heightened metabolic state typical of animals undergoing re-alimentation induced CG [11, 12, 44, 45]. Indeed, this may be a necessary response in order to cope with the typically elevated rate of metabolism associated with greater feed consumption [46], which appears to be a primary driver of whole animal CG [8, 11, 14]. However, further studies are required to assess the metabolic state of the rumen and indeed other metabolic organs in response to both dietary restriction and CG.

In addition to an increase in the expression of genes coding for chaperone and protein folding cellular machinery, up-regulation of genes involved in transcriptional activity was also observed in rumen papillae of animals undergoing CG, again this coincided with greater reticulo-rumen and whole body growth [11]. Overall, genes coding for proteins involved in transcription (*EMG1*, *FOXN1*, *FOXP4*, *INTS3*), splicing (*KHDRBS3*, *PRCC*) and translation (*EIF4G2*, *EIF4G3*, *ELL2*) were up-regulated in rumen papillae of cattle undergoing CG. Transcriptional genes differentially expressed included *EMG1*, which encodes a protein involved in ribosome biogenesis [47], two FoxO proteins which are involved in the regulation of gene transcription [48] and a subunit of the integrator complex of RNA polymerase II (*INTS3*; [49]). Both *EIF4G2* and *EIF4G3* code for proteins involved in the eukaryotic translation initiation factor and function in the recognition of the mRNA cap, and recruitment of mRNA to the ribosome [50]. Up-regulation of *EIF4G2* was also reported in the data of Connor et al. [10] in hepatic tissue of cattle undergoing CG. The elongation factor component *ELL2* was also up-regulated in skeletal muscle tissue of the same cattle used in the current study [25]. This gene codes for a complex which is required to increase the catalytic rate of RNA polymerase II transcription [51]. Genes involved in splicing, and the editing of nascent pre-mRNA [52] were also detected as differentially expressed in rumen papillae of cattle undergoing CG in the current study. These included *KHDRBS3* which functions in the regulation of alternative splicing and influences mRNA splice site selection [53] and *PRCC* which functions in pre-mRNA splicing [54]. Moreover, genes coding for histone proteins (*HIST1H2AC*, *HIST1H2BD*, *HIST1H2BN*, *HISTH2BO*, *HIST2H4A*, *KAT2A*) were also up-regulated during the same time. Histones are proteins that package and order DNA into structural nucleosomes, playing a role in gene regulation [55]. Additionally, *KAT2A* a histone acetlytransferase that functions primarily as a transcriptional activator was also up-regulated in papillae of animals undergoing CG. Genes coding for histone proteins were also detected as up-regulated in skeletal muscle tissue of cattle expressing CG, these included *HIST1H2AC*, *HIST1H2BD* and *KAT2A* [25]. Collectively, these results suggest an increase in gene expression and associated translational and protein folding activity in rumen papillae epithelia during CG and associated feed efficiency in cattle. A similar effect was also reported in rumen epithelium of feed efficient cattle (low-residual feed intake) [56]. This is also apparent in the network presented in Fig 3, where genes associated with metabolism, biochemistry and cellular assembly and organisation were up-regulated. Up-regulation of these cellular processes during rumen papillae CG may be a consequence of a greater nutrient intake during re-alimentation and be necessary for the replenishment of the associated metabolic machinery required for increased digestion and absorption, which ultimately may be contributing to compensatory tissue growth and development, and as stated earlier was also apparent during CG of hepatic tissue of cattle undergoing CG [10, 26].

### Cellular interactions and organisation

Reduced nutrient intake may be consistent with a down-regulation of cellular processes associated with cellular function and organisation [57]. This has previously been reported in skeletal muscle of cattle after diet restriction [25]. Following a period of dietary restriction, genes coding for proteins involved in structural components of ruminal epithelial cells were observed to be down-regulated in RES compared to ADLIB animals. Down-regulation of these genes may be due to a lack of requirement for a large ruminal epithelial surface area as a consequence of a reduction in intake and associated digestive processes in the rumen. Moreover, a reduction in epithelial surface area may allow for a reduction in cellular maintenance requirements in an energetically demanding organ such as the rumen. At the end of Period 1, *CDH2* and *DSG1* were both down-regulated in RES animals compared to ADLIB animals. *CDH2* codes for a cadherin, which are a family of transmembrane proteins involved in cellular adhesion [58]. The encoded protein CDH2, is a calcium dependent cell-cell adhesion glycoprotein [59]. The gene *DSG1* codes for a desmosome protein, which form junctions between certain cell types including epithelial cells [58]. DSG1 is a calcium-binding trans-membrane glycoprotein component of desmosomes in vertebrate epithelial cells [60]. It is involved in maintaining the structural integrity of epithelial cells including rumen epithelium and intermediate filaments mediating cell-cell adhesion [61]. Structural alterations to rumen papillae in response to differences in dietary intake have previously been reported. For example, Steele et al. [62] observed structural adaptations in rumen epithelium when cows were fed a diet consisting primarily of grain. Moreover, in that study lower expression of *DSG1* was reported in response to a high concentrate diet, with expression subsequently greater upon transition to a high forage diet [62]. Additionally, Sun et al. [23] observed reduced rumen epithelial height, width and surface area in goats following a 48 day period of dietary restriction. It is logical to expect that the cumulative surface area of papillae and thus weight of the organ itself reflects the prevailing dietary management of an animal. As a consequence of reduced dietary intake, there may be a decreased necessity for ruminal papillae surface area, which may contribute to the reduction in rumen size, as observed in the current study [11]. In turn, the reduction in rumen size may allow for rumen metabolic rate to be curtailed which in turn could contribute to reduced animal maintenance requirements during dietary restriction.

Conversely though, during re-alimentation induced CG, the corollary was observed in the current study, whereby expression of genes coding for proteins involved in cellular interactions and organisation was greater in papillae of RES animals at the end of Period 2 than at the end of Period 1. Genes involved in cellular adhesion (*ANTXR1*, *CEP97*, *FAT4*, *PCDH12*, *PCDH7*), cellular interactions (*IGCA8*, *NRG1*, *RELN*, *SMAGP*, *THBS4*) and transport (*SLC1A5*, *SLC22A17*, *SLC25A15*, *SLC25A26*, *SLC30A6*, *SLC4A7*, *SLC6A9*, *SLC9A1*, *CACNA1G*, *KCNC4*) were all up-regulated during the CG of ruminal papillae. A similar effect has also been reported in skeletal muscle for the same cattle population used here [25]. Of note, up-regulation of the following genes *PCDH12* and *THBS4* as well as two transporter genes, *SLC22A17* and *SLC25A15* was consistent between the current study for rumen epithelial tissue and our previous study using muscle tissue [25]. Up-regulation of these processes during CG in ruminal epithelial may have reflected a necessary adaptive requirement for cells to cope with the increase in cellular metabolic activity as a consequence of increased nutrient availability. Indeed, the observed improved feed efficiency associated with CG may be through potentially increasing the surface area of rumen papaillae. This hypothesis is further fortified following the results of Sun et al. [23], who showed that rumen papillae height, width and surface area were all lower in goats that had undergone a 6 week period of dietary restriction. However, following a period of CG, full recovery in the morphology of epithelium tissue was observed [23]. Greater expression of genes involved in cellular adhesion and interaction as well as cellular transport proteins in the current study suggest that the structural state of the rumen papillae may play an important role in governing the expression of entire body CG. Indeed, an increase in rumen papillae structure and consequently surface area during re-alimentation may potentially contribute to an improvement in nutrient absorption during periods of accelerated growth, which is consistent with the increase in appetite and feed intake capacity of animals undergoing CG [7, 9, 11, 14]. Moreover, Kong et al. [56] reported up-regulation of genes involved in intracellular adhesion and actin cytoskeleton in the rumen epithelium of feed efficient cattle suggesting that the rumen epithelium may contribute to the enhanced feed efficiency evident during CG. Additionally, restoration of ruminal epithelium may be a necessary requirement in response to re-alimentation in order to cope with the increase in associated metabolic activity concomitant with increased dietary intake which was evident in the animals used in the current study where consumption of feed was greater on a proportional body weight basis in RES compared with ADLIB animals [11]. However, although DEG profiles suggest alterations to rumen papillae surface area in response to both diet restriction and CG, physical measurements, including papillae height, width, crypt depth are necessary to prove this hypothesis in cattle.

### Immune function

Our global gene expression data suggest that the animal’s immune system was also affected by both dietary restriction and subsequent re-alimentation induced CG in rumen epithelial. This was manifested through differential expression of immune related genes namely up-regulation of *IL17A*, and *LBP* in animals undergoing dietary restriction. *IL17A* codes for interleukin 17a, a proinflammatory cytokine [63], whereas *LBP* is involved in host defence against gram negative bacteria and plays a role in innate immune response [64]. Similarly, following a 10-week period of feed restriction, changes in genes regulating immune function and inflammation was apparent in hepatic tissue in the data of Connor et al. [10]. Moreover, Dhahbi et al. [65] reported functional groups of genes to be affected by calorie restriction in mice including those involved in the immune response. Periods of moderate dietary restriction have previously been shown to affect the immune system manifested as an up-regulation of immune genes and an overall greater capacity for immune response following a period of dietary restriction [66–70]. Up-regulation of genes governing the immune response during nutrient restriction may represent a potential protective mechanism against pathological disease. Indeed, a study on rodents showed that the immunological status of rodents offered a restricted feed allowance was superior to that of their non-restricted counterparts [71]. A similar outcome was also apparent in the jejunal epithelial cells of cattle following a period of dietary restriction, whereby *CTSW*, a gene which functions in T-cell cytolytic activity was also up-regulated in cattle that had undergone a period of dietary restriction compared to their *ad libitum* counterparts [24]. Overall, these results suggest that dietary restriction in cattle can elicit a superior immunological status as previously described in other species which may protect against any potential pathological threats to the animal. Alternatively, it has been suggested that the immune response could be involved in nutrient partitioning away from non-essential activities including growth and instead towards activating tissue mobilisation and catabolism [72]. Nutrient partitioning during diet restriction has been widely reported in cattle [11, 12, 44, 73, 74]. When coupled with data from the present study these results indicate that the immune system may be contributing to this observed effect. Indeed in the context of the current study this may be reflective of a change in rumen size and weight in response to a period of dietary restriction.

Immune related genes were subsequently down-regulated in ruminal epithelial during re-alimentation compared with previous dietary restriction. Immune genes down-regulated reflected those involved in inflammation (*BDKRB1*, *CHI3L1*, *HPGD*, *LTA4H*); the complement system (*C5AR2*, *CD59*); cytokines (*CCL19*) and chemokines (*CCL20*, *CXCL12*, *CXCL17*, *CXCL2*, *CXCR4*) as well as others (*LYZ*, immunoagents; *SGSH* and *CYBA*, lysosomal degradation). In the data of Chen et al. [75], *CD59* was also found to be down-regulated in hepatic tissue of feed efficient cattle. Similarly, in the current ruminal papillae study, *CD59* was down-regulated in animals undergoing CG consistent with increased feed efficiency [4, 11]. Moreover, *LTA4H* gene was also down-regulated in the skeletal muscle of our cattle when undergoing CG [25]. Studies in beef cattle divergently selected for feed efficiency have indicated that a large proportion of the variation in efficiency among animals may be attributed to stress or immune related biological pathways [76]. Moreover, Alexandre et al. [77] described down-regulation of genes involved in the immune response in feed efficient cattle, which is consistent with the results of the current study, as during CG the animals in the current study displayed a better feed efficiency potential [11]. Kern et al. [78] recently suggested that a reduction in an animal’s immune response, as described during CG in the current study, may allow for more energy to be directed toward cellular proliferation and growth. As this effect was observed in the current study, it is possible that down-regulation of immune-related genes during re-alimentation may allow for the rapid CG typically observed for the rumen [11]. Alternatively, the same authors suggested that a reduction in the immune response could benefit both intake and gain through a reduction in papillae swelling, which may allow for improved nutrient absorption [78].

## Conclusions

Following a period of dietary restriction, we described evidence for reduced gene expression and cellular interactions in rumen papillae tissue of Holstein Friesian bulls. This was in conjunction with an apparent enhanced immune response potential. During subsequent re-alimentation induced CG, our data suggest that greater nutrient intake is consistent with an up-regulation in transcriptional activity of ruminal epithelial tissue, which may in turn lead to greater nutrient uptake through an increase in papillae surface area and ultimately contribute to increased feed efficiency typical of CG, thus supporting the accelerated growth phenomenon of both the rumen as well as the animal. In contrast to that observed for diet restricted cattle a period of improved feed efficiency was consistent with a reduction in the abundance of transcripts for genes involved in immune response, potentially allowing more energy to be channelled towards growth within the rumen papillae. Our results also suggest that the structural state of the gastrointestinal tract may play an important role in governing feed efficiency, with an increase in rumen papillae surface area during re-alimentation potentially contributing to improvements in nutrient absorption during periods of accelerated growth. The new knowledge generated in this study offers further insights into some of the many molecular processes underlying nutrient restricted and CG states in cattle. However functional studies are now warranted to validate the hypotheses put forward in the current study. Furthermore, our DEG patterns provide baseline data which may be further interrogated and used to identify animals with superior genetic potential for CG and associated feed efficiency.

## Supporting information

S1 Table Genes differentially expressed in rumen epithelium of Holstein Friesian bulls (n = 10) following a 125-day period of restricted feeding at the end of Period 1 relative to *ad libitum*-fed controls (n = 10). (DOCX)

S2 Table Genes differentially expressed in rumen epithelium of Holstein Friesian bulls (n = 10) following a 55-day period of re-alimentation and compensatory growth in Period 2 relative to animals fed a restricted diet for 125 days at the end of Period 1(n = 10). (DOCX)

S3 Table Networks generated from gene expression data of restricted versus *ad libitum* fed bulls by IPA. (DOCX)

S4 Table Networks generated from gene expression data of compensating versus restricted fed bulls by IPA. (DOCX)
