# Supplementary material for: Effect of dietary restriction and subsequent re-alimentation on the transcriptional profile of bovine ruminal epithelium
Source: PLoS One. 2017 May 17;12(5):e0177852. doi: 10.1371/journal.pone.0177852 (PMC5435337; doi:10.1371/journal.pone.0177852)
Supplement: S1 Table — (DOCX) [file pone.0177852.s001.docx]

**S1 Table.** Genes differentially expressed in rumen epithelium of Holstein Friesian bulls (n = 10) following a 125-day period of restricted feeding at the end of Period 1 relative to *ad libitum*-fed controls (n = 10)

| Gene | Entrez Gene Name | Fold Change |
| --- | --- | --- |
| symbol |  |  |
| *ALOX15B* | Arachidonate 15-lipoxygenase, type B | -1.759 |
| *ALOX5AP* | Arachidonate 5-lipoxygenase activating protein | -1.748 |
| *ANGPTL1* | Angiopoietin like 1 | -2.227 |
| *BEX2* | Brain expressed X-linked 2 | 1.635 |
| *BFAR* | Bifunctional apoptosis regulator | 1.329 |
| *BMPR1B* | Bone morphogenetic protein receptor type 1B | 2.145 |
| *C4orf19* | Chromosome 4 open reading frame 19 | 2.507 |
| *CCDC8* | Coiled-coil domain containing 8 | 1.711 |
| *CCSER1* | Coiled-coil serine rich protein 1 | 2.215 |
| *CD302* | CD302 molecule | 1.432 |
| *CDH2* | Cadherin 2 | -2.895 |
| *CHPT1* | Choline phosphotransferase 1 | 1.544 |
| *CIRBP* | Cold inducible RNA binding protein | 1.521 |
| *CRYAB* | Crystallin alpha B | -1.708 |
| *DHX58* | DEXH-box helicase 58 | 1.315 |
| *DSG1* | Desmoglein 1 | -4.632 |
| *DUSP6* | Dual specificity phosphatase 6 | -1.444 |
| *ELF3* | E74 like ETS transcription factor 3 | 1.899 |
| *FCER1A* | Fc fragment of IgE receptor Ia | -1.912 |
| *FERMT2* | Fermitin family member 2 | 1.456 |
| *GAN* | Gigaxonin | -1.603 |
| *GMDS* | GDP-mannose 4,6-dehydratase | 1.457 |
| *GPAT3* | Glycerol-3-phosphate acyltransferase 3 | -1.681 |
| *GPR143* | G protein-coupled receptor 143 | -2.439 |
| *HERC3* | HECT and RLD domain containing E3 ubiquitin protein ligase 3 | 1.418 |
| *HIST1H2BD* | Histone cluster 1, H2bd | -1.608 |
| *HIST1H2BN* | Histone cluster 1, H2bn | -2.036 |
| *HSPA4L* | Heat shock protein family A (Hsp70) member 4 like | -1.482 |
| *HSPB8* | Heat shock protein family B (small) member 8 | -1.466 |
| *HSPH1* | Heat shock protein family H (Hsp110) member 1 | -1.628 |
| *IL17A* | Interleukin 17A | 3.707 |
| *INPP4B* | Inositol polyphosphate-4-phosphatase type II B | -1.619 |
| *KCNE4* | Potassium voltage-gated channel subfamily E regulatory subunit 4 | 2.434 |
| *KIAA1755* | KIAA1755 | 1.723 |
| *LBP* | Lipopolysaccharide binding protein | 1.74 |
| *LGALS4* | Lectin, galactoside binding soluble 4 | -1.504 |
| *LRMP* | Lymphoid restricted membrane protein | 2.81 |
| *LRRC66* | Leucine rich repeat containing 66 | 2.333 |
| *MRC2* | Mannose receptor C type 2 | -1.482 |
| *MTUS1* | Microtubule associated tumor suppressor 1 | 1.286 |
| *NAT9* | N-acetyltransferase 9 (putative) | 1.304 |
| *NR4A1* | Nuclear receptor subfamily 4 group A member 1 | 2.564 |
| *NTRK2* | Neurotrophic receptor tyrosine kinase 2 | 1.823 |
| *PAMR1* | Peptidase domain containing associated with muscle regeneration 1 | -2.006 |
| *PANK3* | Pantothenate kinase 3 | -1.477 |
| *PGM5* | Phosphoglucomutase 5 | 1.395 |
| *PIK3C2G* | Phosphatidylinositol-4-phosphate 3-kinase catalytic subunit type 2 gamma | 1.735 |
| *PRDX6* | Peroxiredoxin 6 | -1.727 |
| *RAB20* | RAB20, member RAS oncogene family | 2.282 |
| *RBM3* | RNA binding motif (RNP1, RRM) protein 3 | 1.577 |
| *SATB1* | SATB homeobox 1 | 2.354 |
| *SCUBE1* | Signal peptide, CUB domain and EGF like domain containing 1 | 2.189 |
| *SHROOM1* | Shroom family member 1 | 2.011 |
| *SNRNP25* | Small nuclear ribonucleoprotein U11/U12 subunit 25 | -1.386 |
| *SOCS2* | Suppressor of cytokine signaling 2 | 1.703 |
| *TGM2* | Transglutaminase 2 | 2.201 |
| *TMEM145* | Transmembrane protein 145 | 1.778 |
| *USP20* | Ubiquitin specific peptidase 20 | 1.486 |
| *WNT2B* | Wnt family member 2B | 2.001 |
| *YOD1* | YOD1 deubiquitinase | -2.341 |
| *ZC3H12A* | Zinc finger CCCH-type containing 12A | 1.748 |
| *ZCWPW1* | Zinc finger CW-type and PWWP domain containing 1 | 1.667 |

^1^ Fold changes are up or down in restricted fed animals compared to *ad libitum* control animals
