# Supplementary material for: Effect of dietary restriction and subsequent re-alimentation on the transcriptional profile of bovine ruminal epithelium
Source: PLoS One. 2017 May 17;12(5):e0177852. doi: 10.1371/journal.pone.0177852 (PMC5435337; doi:10.1371/journal.pone.0177852)
Supplement: S2 Table — (DOCX) [file pone.0177852.s002.docx]

**S2 Table.** Genes differentially expressed in rumen epithelium of Holstein Friesian bulls (n = 10) following a 55-day period of re-alimentation and compensatory growth in Period 2 relative to animals fed a restricted diet for 125 days at the end of Period 1(n = 10)

| Gene | Entrez Gene Name | Fold Change |
| --- | --- | --- |
| symbol |  |  |
| *A2M* | Alpha-2-macroglobulin | -1.509 |
| *AADACL3* | Arylacetamide deacetylase-like 3 | 2.141 |
| *AASS* | Aminoadipate-semialdehyde synthase | -1.784 |
| *ABI3BP* | ABI family member 3 binding protein | -1.707 |
| *ACAD9* | Acyl-CoA dehydrogenase family member 9 | -1.295 |
| *ACADVL* | Acyl-CoA dehydrogenase, very long chain | 1.288 |
| *ACKR4* | Atypical chemokine receptor 4 | -2.348 |
| *ACOT7* | Acyl-CoA thioesterase 7 | 1.603 |
| *ACOT9* | Acyl-CoA thioesterase 9 | -1.402 |
| *ACPP* | Acid phosphatase, prostate | -2.409 |
| *ADAMTS4* | ADAM metallopeptidase with thrombospondin type 1 motif 4 | -1.841 |
| *ADCY5* | Adenylate cyclase 5 | -2.012 |
| *ADIRF* | Adipogenesis regulatory factor | -1.751 |
| *AHSA1* | AHA1, activator of heat shock 90kDa protein ATPase homolog 1 (yeast) | 1.491 |
| *AIG1* | Androgen-induced 1 | -1.324 |
| *AKR1C3* | Aldo-keto reductase family 1, member C3 | -2.196 |
| *AKT1S1* | AKT1 substrate 1 | 1.314 |
| *ALDH1A2* | Aldehyde dehydrogenase 1 family member A2 | 1.899 |
| *ALDH2* | Aldehyde dehydrogenase 2 family (mitochondrial) | -1.554 |
| *ALOX15B* | Arachidonate 15-lipoxygenase, type B | 2.395 |
| *AMDHD2* | Amidohydrolase domain containing 2 | -1.339 |
| *AMPD2* | Adenosine monophosphate deaminase 2 | 1.389 |
| *ANGPT2* | Angiopoietin 2 | 1.794 |
| *ANGPT4* | Angiopoietin 4 | -1.707 |
| *ANGPTL1* | Angiopoietin like 1 | 2.703 |
| *ANPEP* | Alanyl aminopeptidase, membrane | 1.641 |
| *ANTXR1* | Anthrax toxin receptor 1 | 1.347 |
| *AP4B1* | Adaptor related protein complex 4 beta 1 subunit | 1.288 |
| *APMAP* | Adipocyte plasma membrane associated protein | -1.291 |
| *APOE* | Apolipoprotein E | -2.053 |
| *APTX* | Aprataxin | -1.262 |
| *ARNTL* | Aryl hydrocarbon receptor nuclear translocator like | -1.35 |
| *ARSK* | Arylsulfatase family member K | -1.595 |
| *AS3MT* | Arsenite methyltransferase | -1.296 |
| *ASPN* | Asporin | 2.092 |
| *ATP5G1* | ATP synthase, H+ transporting, mitochondrial Fo complex subunit C1 (subunit 9) | 1.289 |
| *BAG2* | BCL2 associated athanogene 2 | 1.432 |
| *BAG3* | BCL2 associated athanogene 3 | 1.754 |
| *BCL6* | B-cell CLL/lymphoma 6 | 1.761 |
| *BDKRB1* | Bradykinin receptor B1 | -1.971 |
| *BEX2* | Brain expressed X-linked 2 | -2.067 |
| *BMP2* | Bone morphogenetic protein 2 | 1.646 |
| *BMPR1B* | Bone morphogenetic protein receptor type 1B | -5.406 |
| *BMX* | BMX non-receptor tyrosine kinase | -1.932 |
| *BPHL* | Biphenyl hydrolase-like (serine hydrolase) | -1.385 |
| *BTC* | Betacellulin | -2.117 |
| *C10orf107* | Chromosome 10 open reading frame 107 | -1.809 |
| *C12orf45* | Chromosome 12 open reading frame 45 | -1.475 |
| *C2orf54* | Chromosome 2 open reading frame 54 | -1.629 |
| *C4orf19* | Chromosome 4 open reading frame 19 | -2.145 |
| *C5AR2* | Complement component 5a receptor 2 | -1.605 |
| *CA13* | Carbonic anhydrase 13 | -1.478 |
| *CA5B* | Carbonic anhydrase 5B | -1.457 |
| *CACNA1G* | Calcium voltage-gated channel subunit alpha1 G | 1.975 |
| *CACYBP* | Calcyclin binding protein | 1.466 |
| *CAPG* | Capping actin protein, gelsolin like | -1.513 |
| *CCDC8* | Coiled-coil domain containing 8 | -1.568 |
| *CCL19* | C-C motif chemokine ligand 19 | -4.237 |
| *CCL20* | C-C motif chemokine ligand 20 | -2.965 |
| *CCNB2* | Cyclin B2 | -1.44 |
| *CCNF* | Cyclin F | -1.55 |
| *CCSER1* | Coiled-coil serine rich protein 1 | -1.951 |
| *CCT2* | Chaperonin containing TCP1 subunit 2 | 1.262 |
| *CD300LG* | CD300 molecule like family member g | -1.949 |
| *CD302* | CD302 molecule | -1.79 |
| *CD3G* | CD3g molecule | 2.096 |
| *CD59* | CD59 molecule | -1.458 |
| *CD63* | CD63 molecule | -1.263 |
| *CDH2* | Cadherin 2 | 2.234 |
| *CEP97* | Centrosomal protein 97 | 1.461 |
| *CH25H* | Cholesterol 25-hydroxylase | -1.859 |
| *CHI3L1* | Chitinase 3 like 1 | -3.03 |
| *CHI3L2* | Chitinase 3 like 2 | -3.447 |
| *CHORDC1* | Cysteine and histidine rich domain containing 1 | 1.607 |
| *CHP2* | Calcineurin like EF-hand protein 2 | 1.625 |
| *CHPT1* | Choline phosphotransferase 1 | -1.744 |
| *CIRBP* | Cold inducible RNA binding protein | -1.833 |
| *CKM* | Creatine kinase, M-type | 1.955 |
| *CLDN23* | Claudin 23 | -2.435 |
| *CLEC3B* | C-type lectin domain family 3 member B | -1.814 |
| *CLPB* | ClpB homolog, mitochondrial AAA ATPase chaperonin | 1.358 |
| *Cmah* | Cytidine monophospho-N-acetylneuraminic acid hydroxylase | -1.609 |
| *CNN3* | Calponin 3 | -1.279 |
| *COL12A1* | Collagen type XII alpha 1 | 1.756 |
| *COL4A1* | Collagen type IV alpha 1 | 1.944 |
| *COL4A2* | Collagen type IV alpha 2 | 1.859 |
| *COMTD1* | Catechol-O-methyltransferase domain containing 1 | 1.358 |
| *COX10* | COX10 heme A:farnesyltransferase cytochrome c oxidase assembly factor | 1.278 |
| *Cox7c* | Cytochrome c oxidase subunit VIIc | -1.565 |
| *CPZ* | Carboxypeptidase Z | -2.065 |
| *CRYAB* | Crystallin alpha B | 2.88 |
| *CTSB* | Cathepsin B | -1.275 |
| *CTTNBP2* | Cortactin binding protein 2 | -2.303 |
| *CXCL12* | C-X-C motif chemokine ligand 12 | -2.189 |
| *CXCL17* | C-X-C motif chemokine ligand 17 | -2.691 |
| *CXCL2* | C-X-C motif chemokine ligand 2 | -2.957 |
| *CXCR4* | C-X-C motif chemokine receptor 4 | -1.646 |
| *CYBA* | Cytochrome b-245 alpha chain | -1.418 |
| *DGAT2* | Diacylglycerol O-acyltransferase 2 | 2.027 |
| *DHRS11* | Dehydrogenase/reductase (SDR family) member 11 | 1.333 |
| *DHRS12* | Dehydrogenase/reductase (SDR family) member 12 | -1.513 |
| *DHRS9* | Dehydrogenase/reductase (SDR family) member 9 | -2.707 |
| *DHX58* | DEXH-box helicase 58 | -1.39 |
| *DLX3* | Distal-less homeobox 3 | 1.899 |
| *DNAJB4* | DnaJ heat shock protein family (Hsp40) member B4 | 1.304 |
| *DOCK3* | Dedicator of cytokinesis 3 | -1.608 |
| *DPP9* | Dipeptidyl peptidase 9 | 1.354 |
| *DPY19L1* | Dpy-19 like 1 (C. elegans) | -1.501 |
| *DSG1* | Desmoglein 1 | 4.647 |
| *DUOX1* | Dual oxidase 1 | 1.394 |
| *DUT* | Deoxyuridine triphosphatase | -1.369 |
| *DYRK3* | Dual specificity tyrosine phosphorylation regulated kinase 3 | 1.312 |
| *EDAR* | Ectodysplasin A receptor | -1.353 |
| *EDN3* | Endothelin 3 | -2.098 |
| *EFEMP1* | EGF containing fibulin-like extracellular matrix protein 1 | -2.171 |
| *EFS* | Embryonal Fyn-associated substrate | 1.405 |
| *EHHADH* | Enoyl-CoA, hydratase/3-hydroxyacyl CoA dehydrogenase | -1.476 |
| *EIF4G2* | Eukaryotic translation initiation factor 4 gamma 2 | 1.25 |
| *EIF4G3* | Eukaryotic translation initiation factor 4 gamma 3 | 1.252 |
| *ELF3* | E74 like ETS transcription factor 3 | -2.004 |
| *ELL2* | Elongation factor for RNA polymerase II 2 | 1.423 |
| *ELOVL4* | ELOVL fatty acid elongase 4 | 2.589 |
| *EMG1* | EMG1 N1-specific pseudouridine methyltransferase | 1.267 |
| *ENG* | Endoglin | 1.372 |
| *ENPEP* | Glutamyl aminopeptidase | 2.479 |
| *EPB41L3* | Erythrocyte membrane protein band 4.1 like 3 | 1.535 |
| *EPS8* | Epidermal growth factor receptor pathway substrate 8 | -1.381 |
| *ETFDH* | Electron transfer flavoprotein dehydrogenase | -1.286 |
| *ETNPPL* | Ethanolamine-phosphate phospho-lyase | -3.766 |
| *FADS3* | Fatty acid desaturase 3 | 2.251 |
| *FAM107B* | Family with sequence similarity 107 member B | -1.511 |
| *FAM3B* | Family with sequence similarity 3 member B | -1.53 |
| *FARP1* | FERM, ARH/RhoGEF and pleckstrin domain protein 1 | -1.463 |
| *FAT4* | FAT atypical cadherin 4 | 1.609 |
| *FBP2* | Fructose-bisphosphatase 2 | -1.631 |
| *FBXO17* | F-box protein 17 | -1.625 |
| *FBXO47* | F-box protein 47 | -1.87 |
| *FCER1A* | Fc fragment of IgE receptor Ia | 1.799 |
| *FDFT1* | Farnesyl-diphosphate farnesyltransferase 1 | -1.329 |
| *FERMT2* | Fermitin family member 2 | -1.507 |
| *FGD2* | FYVE, RhoGEF and PH domain containing 2 | -1.563 |
| *FGFR2* | Fibroblast growth factor receptor 2 | -1.392 |
| *FKBP4* | FK506 binding protein 4 | 1.369 |
| *FOXN1* | Forkhead box N1 | 2.581 |
| *FOXP4* | Forkhead box P4 | 1.403 |
| *FRRS1* | Ferric chelate reductase 1 | -1.541 |
| *FUT2* | Fucosyltransferase 2 | 1.962 |
| *GABARAPL1* | GABA type A receptor associated protein like 1 | -1.389 |
| *GABBR1* | Gamma-aminobutyric acid type B receptor subunit 1 | -1.367 |
| *GALNT16* | Polypeptide N-acetylgalactosaminyltransferase 16 | -2.087 |
| *GALNT8* | Polypeptide N-acetylgalactosaminyltransferase 8 | 2.61 |
| *GAS2L1* | Growth arrest specific 2 like 1 | 1.466 |
| *GCLM* | Glutamate-cysteine ligase modifier subunit | 1.46 |
| *GFPT2* | Glutamine-fructose-6-phosphate transaminase 2 | -3.021 |
| *GHR* | Growth hormone receptor | -2.483 |
| *GINS2* | GINS complex subunit 2 | -1.366 |
| *GK5* | Glycerol kinase 5 (putative) | -1.388 |
| *GLB1* | Galactosidase beta 1 | -1.379 |
| *GLIPR1* | GLI pathogenesis related 1 | -1.502 |
| *GLP1R* | Glucagon like peptide 1 receptor | 1.448 |
| *GNAL* | G protein subunit alpha L | 1.557 |
| *GPAT3* | Glycerol-3-phosphate acyltransferase 3 | 1.493 |
| *GPC3* | Glypican 3 | 1.78 |
| *GPR143* | G protein-coupled receptor 143 | 4.169 |
| *GPRC5B* | G protein-coupled receptor class C group 5 member B | -1.564 |
| *GPSM2* | G-protein signaling modulator 2 | -1.257 |
| *GSPT1* | G1 to S phase transition 1 | 1.265 |
| *GSTM1* | Glutathione S-transferase mu 1 | 1.451 |
| *GSTM4* | Glutathione S-transferase mu 4 | 1.306 |
| *HABP4* | Hyaluronan binding protein 4 | 1.675 |
| *HAUS4* | HAUS augmin like complex subunit 4 | -1.353 |
| *HERC3* | HECT and RLD domain containing E3 ubiquitin protein ligase 3 | -1.513 |
| *HIST1H2AC* | Histone cluster 1, H2ac | 2.538 |
| *HIST1H2BD* | Histone cluster 1, H2bd | 1.595 |
| *HIST1H2BO* | Histone cluster 1, H2bo | 1.331 |
| *HIST1H4J* | Histone cluster 1, H4j | -2.035 |
| *HIST2H4A* | Histone cluster 2, H4a | 1.634 |
| *HLCS* | Holocarboxylase synthetase | 1.263 |
| *HMCN1* | Hemicentin 1 | 1.676 |
| *HMCN2* | Hemicentin 2 | 1.572 |
| *HMGB2* | High mobility group box 2 | -1.42 |
| *HPGD* | Hydroxyprostaglandin dehydrogenase 15-(NAD) | -1.674 |
| *HPSE* | Heparanase | 1.448 |
| *HSP90AA1* | Heat shock protein 90kDa alpha family class A member 1 | 1.697 |
| *HSP90AB1* | Heat shock protein 90kDa alpha family class B member 1 | 1.284 |
| *HSPA4L* | Heat shock protein family A (Hsp70) member 4 like | 1.636 |
| *HSPA8* | Heat shock protein family A (Hsp70) member 8 | 1.543 |
| *HSPB1* | Heat shock protein family B (small) member 1 | 1.422 |
| *HSPB8* | Heat shock protein family B (small) member 8 | 1.739 |
| *HSPD1* | Heat shock protein family D (Hsp60) member 1 | 1.306 |
| *HSPE1* | Heat shock protein family E (Hsp10) member 1 | 1.375 |
| *HSPH1* | Heat shock protein family H (Hsp110) member 1 | 2.557 |
| *ICA1* | Islet cell autoantigen 1 | -1.804 |
| *ID2* | Inhibitor of DNA binding 2, HLH protein | 1.506 |
| *IGFBP5* | Insulin like growth factor binding protein 5 | 1.605 |
| *INTS3* | Integrator complex subunit 3 | 1.28 |
| *ISL1* | ISL LIM homeobox 1 | 1.8 |
| *ISLR* | Immunoglobulin superfamily containing leucine-rich repeat | -2.608 |
| *ISM1* | Isthmin 1, angiogenesis inhibitor | 1.486 |
| *ITGA8* | Integrin subunit alpha 8 | 1.746 |
| *ITGB2* | Integrin subunit beta 2 | -2.263 |
| *ITIH5* | Inter-alpha-trypsin inhibitor heavy chain family member 5 | 1.439 |
| *KAT2A* | Lysine acetyltransferase 2A | 1.25 |
| *KCNC4* | Potassium voltage-gated channel subfamily C member 4 | 1.492 |
| *KCNMA1* | Potassium calcium-activated channel subfamily M alpha 1 | -2.311 |
| *KCNMB1* | Potassium calcium-activated channel subfamily M regulatory beta subunit 1 | -1.563 |
| *KHDRBS3* | KH domain containing, RNA binding, signal transduction associated 3 | 1.488 |
| *KIAA0922* | KIAA0922 | -1.268 |
| *KIF20A* | Kinesin family member 20A | -1.39 |
| *KIF21A* | Kinesin family member 21A | -1.402 |
| *KIF4A* | Kinesin family member 4A | -1.32 |
| *KPTN* | Kaptin (actin binding protein) | 1.257 |
| *KRT14* | Keratin 14 | 1.546 |
| *KRT75* | Keratin 75 | 2.403 |
| *KRT78* | Keratin 78 | -1.353 |
| *LGALS3* | Lectin, galactoside binding soluble 3 | -1.628 |
| *LIMCH1* | LIM and calponin homology domains 1 | 1.591 |
| *LIX1* | Limb and CNS expressed 1 | -7.364 |
| *LMOD1* | Leiomodin 1 | -1.47 |
| *LOX* | Lysyl oxidase | 1.714 |
| *LPL* | Lipoprotein lipase | -4.523 |
| *LRMP* | Lymphoid restricted membrane protein | -2.746 |
| *LRRC1* | Leucine rich repeat containing 1 | -1.254 |
| *LRRC3B* | Leucine rich repeat containing 3B | -2.367 |
| *LRRC66* | Leucine rich repeat containing 66 | -1.988 |
| *LTA4H* | Leukotriene A4 hydrolase | -1.276 |
| *LYPD6* | LY6/PLAUR domain containing 6 | -1.659 |
| *LYRM1* | LYR motif containing 1 | -1.571 |
| *LYZ* | Lysozyme | -3.144 |
| *MAL* | Mal T-cell differentiation protein | -1.425 |
| *MAOB* | Monoamine oxidase B | 2.989 |
| *MARCKS* | Myristoylated alanine rich protein kinase C substrate | -1.369 |
| *MAST2* | Microtubule associated serine/threonine kinase 2 | 1.339 |
| *MCOLN1* | Mucolipin 1 | -1.277 |
| *MCRIP2* | MAPK regulated co-repressor interacting protein 2 | 1.34 |
| *MDN1* | Midasin AAA ATPase 1 | 1.386 |
| *ME2* | Malic enzyme 2 | 1.327 |
| *METTL23* | Methyltransferase like 23 | 1.394 |
| *MIA* | Melanoma inhibitory activity | 1.823 |
| *MMACHC* | Methylmalonic aciduria (cobalamin deficiency) cblC type, with homocystinuria | 1.397 |
| *MMP13* | Matrix metallopeptidase 13 | 1.651 |
| *MMP2* | Matrix metallopeptidase 2 | 1.67 |
| *MMP28* | Matrix metallopeptidase 28 | 1.417 |
| *MND1* | Meiotic nuclear divisions 1 | -1.547 |
| *MOG* | Myelin oligodendrocyte glycoprotein | -1.492 |
| *MRC2* | Mannose receptor C type 2 | 1.733 |
| *MSS51* | MSS51 mitochondrial translational activator | -1.415 |
| *MTHFR* | Methylenetetrahydrofolate reductase (NAD(P)H) | -1.449 |
| *MT-ND5* | NADH dehydrogenase, subunit 5 (complex I) | -1.582 |
| *MT-ND6* | NADH dehydrogenase, subunit 6 (complex I) | -1.927 |
| *MTSS1* | Metastasis suppressor 1 | -1.328 |
| *MTUS1* | Microtubule associated tumor suppressor 1 | -1.38 |
| *MX2* | MX dynamin like GTPase 2 | -1.832 |
| *MYLIP* | Myosin regulatory light chain interacting protein | 1.31 |
| *MYO18B* | Myosin XVIIIB | 3.328 |
| *NAA50* | N(alpha)-acetyltransferase 50, NatE catalytic subunit | 1.319 |
| *NABP1* | Nucleic acid binding protein 1 | -1.337 |
| *NDRG2* | NDRG family member 2 | -1.462 |
| *NDUFA4L2* | NADH dehydrogenase (ubiquinone) 1 alpha subcomplex, 4-like 2 | 1.905 |
| *NID1* | Nidogen 1 | 1.392 |
| *NIPSNAP3A* | Nipsnap homolog 3A | -1.301 |
| *NQO2* | NAD(P)H quinone dehydrogenase 2 | -1.816 |
| *NR3C2* | Nuclear receptor subfamily 3 group C member 2 | -1.754 |
| *NRG1* | Neuregulin 1 | 1.512 |
| *NTRK2* | Neurotrophic receptor tyrosine kinase 2 | -2.512 |
| *OCLN* | Occludin | -1.291 |
| *OSBPL9* | Oxysterol binding protein like 9 | -1.322 |
| *P2RY1* | Purinergic receptor P2Y1 | 1.449 |
| *P2RY2* | Purinergic receptor P2Y2 | -1.87 |
| *P4HA1* | Prolyl 4-hydroxylase subunit alpha 1 | 1.391 |
| *P4HA2* | Prolyl 4-hydroxylase subunit alpha 2 | 1.747 |
| *PAFAH1B3* | Platelet activating factor acetylhydrolase 1b catalytic subunit 3 | -1.255 |
| *PALM* | Paralemmin | -1.627 |
| *PAM* | Peptidylglycine alpha-amidating monooxygenase | 1.343 |
| *PAMR1* | Peptidase domain containing associated with muscle regeneration 1 | 2.133 |
| *PARD6A* | Par-6 family cell polarity regulator alpha | 1.816 |
| *PCDH12* | Protocadherin 12 | 1.715 |
| *PCDH7* | Protocadherin 7 | 1.666 |
| *PCOLCE2* | Procollagen C-endopeptidase enhancer 2 | -2.058 |
| *PDE6C* | Phosphodiesterase 6C | -2.684 |
| *PDGFA* | Platelet derived growth factor subunit A | -1.696 |
| *PDGFC* | Platelet derived growth factor C | -1.437 |
| *PDK4* | Pyruvate dehydrogenase kinase 4 | -1.937 |
| *PER1* | Period circadian clock 1 | -1.862 |
| *PIBF1* | Progesterone immunomodulatory binding factor 1 | 1.302 |
| *PIK3C2G* | Phosphatidylinositol-4-phosphate 3-kinase catalytic subunit type 2 gamma | -2.065 |
| *PLA2G4F* | Phospholipase A2 group IVF | -1.475 |
| *PLBD1* | Phospholipase B domain containing 1 | -1.577 |
| *PLCG2* | Phospholipase C gamma 2 | -1.534 |
| *PLCH1* | Phospholipase C eta 1 | -1.46 |
| *PLVAP* | Plasmalemma vesicle associated protein | 1.287 |
| *PLXDC2* | Plexin domain containing 2 | -1.382 |
| *POSTN* | Periostin, osteoblast specific factor | -1.518 |
| *PPID* | Peptidylprolyl isomerase D | 1.392 |
| *PPP3CC* | Protein phosphatase 3 catalytic subunit gamma | -1.287 |
| *PRC1* | Protein regulator of cytokinesis 1 | -1.334 |
| *PRCC* | Papillary renal cell carcinoma (translocation-associated) | 1.253 |
| *PRDX6* | Peroxiredoxin 6 | 2.081 |
| *PRKD1* | Protein kinase D1 | -1.388 |
| *PROCR* | Protein C receptor | -1.39 |
| *PRSS22* | Protease, serine 22 | -1.695 |
| *PRSS35* | Protease, serine 35 | -2.628 |
| *PSD* | Pleckstrin and Sec7 domain containing | 1.815 |
| *PSPC1* | Paraspeckle component 1 | -1.298 |
| *PTPRS* | Protein tyrosine phosphatase, receptor type S | 1.294 |
| *PYURF* | PIGY upstream reading frame | -1.28 |
| *QPCT* | Glutaminyl-peptide cyclotransferase | -1.689 |
| *R3HDM1* | R3H domain containing 1 | 1.307 |
| *RAB20* | RAB20, member RAS oncogene family | -2.243 |
| *RASA4* | RAS p21 protein activator 4 | -1.902 |
| *RB1* | Retinoblastoma 1 | 1.299 |
| *RBM3* | RNA binding motif (RNP1, RRM) protein 3 | -1.864 |
| *RBP2* | Retinol binding protein 2 | -1.559 |
| *RCAN2* | Regulator of calcineurin 2 | -1.758 |
| *RELN* | Reelin | 1.647 |
| *RENBP* | Renin binding protein | -1.312 |
| *RGS1* | Regulator of G-protein signaling 1 | -2.397 |
| *RIBC1* | RIB43A domain with coiled-coils 1 | -1.575 |
| *RMND5B* | Required for meiotic nuclear division 5 homolog B | -1.264 |
| *RNASE1* | Ribonuclease A family member 1, pancreatic | -5.678 |
| *RNASEH2A* | Ribonuclease H2 subunit A | -1.414 |
| *RNASEL* | Ribonuclease L | -1.592 |
| *RNASET2* | Ribonuclease T2 | -1.525 |
| *ROBO4* | Roundabout guidance receptor 4 | 1.523 |
| *ROR2* | Receptor tyrosine kinase like orphan receptor 2 | -1.986 |
| *RORC* | RAR related orphan receptor C | -1.65 |
| *RPL36A* | Ribosomal protein L36a | -1.457 |
| *RPS3A* | Ribosomal protein S3A | -1.655 |
| *RRP1B* | Ribosomal RNA processing 1B | -1.293 |
| *RTP3* | Receptor (chemosensory) transporter protein 3 | 1.317 |
| *RUNX2* | Runt related transcription factor 2 | 1.825 |
| *S1PR1* | Sphingosine-1-phosphate receptor 1 | -1.381 |
| *SAT1* | Spermidine/spermine N1-acetyltransferase 1 | -1.319 |
| *SATB1* | SATB homeobox 1 | -2.191 |
| *SBSN* | Suprabasin | 3.623 |
| *SCARA5* | Scavenger receptor class A member 5 | -4.802 |
| *SCCPDH* | Saccharopine dehydrogenase (putative) | -1.281 |
| *SCIN* | Scinderin | -3.727 |
| *SDK2* | Sidekick cell adhesion molecule 2 | 1.402 |
| *SERPINH1* | Serpin family H member 1 | 1.573 |
| *SFRP2* | Secreted frizzled-related protein 2 | -1.823 |
| *SFTPC* | Surfactant protein C | -1.991 |
| *SGK223* | Homolog of rat pragma of Rnd2 | 1.871 |
| *SGSH* | N-sulfoglucosamine sulfohydrolase | -1.261 |
| *SH2D4A* | SH2 domain containing 4A | -1.335 |
| *SH3BGRL* | SH3 domain binding glutamate rich protein like | -1.526 |
| *SH3PXD2A* | SH3 and PX domains 2A | 1.307 |
| *SHROOM1* | Shroom family member 1 | -2.209 |
| *SIK2* | Salt inducible kinase 2 | 1.277 |
| *SKIL* | SKI-like proto-oncogene | 1.513 |
| *SLC1A5* | Solute carrier family 1 member 5 | 1.342 |
| *SLC22A17* | Solute carrier family 22 member 17 | 2.296 |
| *SLC25A15* | Solute carrier family 25 member 15 | 1.276 |
| *SLC25A26* | Solute carrier family 25 member 26 | 1.251 |
| *SLC30A6* | Solute carrier family 30 member 6 | 1.295 |
| *SLC46A3* | Solute carrier family 46 member 3 | -1.404 |
| *SLC4A7* | Solute carrier family 4 member 7 | 1.397 |
| *SLC6A9* | Solute carrier family 6 member 9 | 1.349 |
| *SLC9A1* | Solute carrier family 9 member A1 | 1.318 |
| *SLITRK6* | SLIT and NTRK like family member 6 | 2.343 |
| *SMAGP* | Small cell adhesion glycoprotein | 1.303 |
| *SNRNP25* | Small nuclear ribonucleoprotein U11/U12 subunit 25 | 1.512 |
| *SOCS2* | Suppressor of cytokine signaling 2 | -1.57 |
| *SPA17* | Sperm autoantigenic protein 17 | -1.508 |
| *SPAG5* | Sperm associated antigen 5 | -1.412 |
| *SPATA7* | Spermatogenesis associated 7 | -1.429 |
| *SPATS2L* | Spermatogenesis associated serine rich 2 like | -1.355 |
| *SPC24* | SPC24, NDC80 kinetochore complex component | -1.446 |
| *SPON2* | Spondin 2 | -1.737 |
| *SPRED1* | Sprouty related EVH1 domain containing 1 | 1.414 |
| *SQRDL* | Sulfide quinone reductase-like (yeast) | 1.275 |
| *SRPK2* | SRSF protein kinase 2 | -1.406 |
| *ST3GAL4* | ST3 beta-galactoside alpha-2,3-sialyltransferase 4 | -1.967 |
| *STAB1* | Stabilin 1 | 1.354 |
| *STIP1* | Stress induced phosphoprotein 1 | 1.436 |
| *STYX* | Serine/threonine/tyrosine interacting protein | 1.392 |
| *SUGCT* | Succinyl-CoA:glutarate-CoA transferase | -1.681 |
| *Sult1a1* | Sulfotransferase family 1A, phenol-preferring, member 1 | -1.379 |
| *SYNGAP1* | Synaptic Ras GTPase activating protein 1 | 1.447 |
| *SYT3* | Synaptotagmin 3 | 2.029 |
| *TANC1* | Tetratricopeptide repeat, ankyrin repeat and coiled-coil containing 1 | -1.276 |
| *TBC1D2* | TBC1 domain family member 2 | -1.345 |
| *TDRD7* | Tudor domain containing 7 | -1.273 |
| *TGFBI* | Transforming growth factor beta induced | 1.602 |
| *TGM2* | Transglutaminase 2 | -1.823 |
| *THAP2* | THAP domain containing, apoptosis associated protein 2 | 1.368 |
| *THBS2* | Thrombospondin 2 | 1.667 |
| *THBS4* | Thrombospondin 4 | 1.818 |
| *THOP1* | Thimet oligopeptidase 1 | 1.425 |
| *TM7SF2* | Transmembrane 7 superfamily member 2 | 1.329 |
| *TMEM119* | Transmembrane protein 119 | 1.552 |
| *TMEM131* | Transmembrane protein 131 | 1.275 |
| *TMEM144* | Transmembrane protein 144 | -1.539 |
| *TNS4* | Tensin 4 | 1.362 |
| *TRIP10* | Thyroid hormone receptor interactor 10 | -1.466 |
| *TSSK1B* | Testis specific serine kinase 1B | 1.699 |
| *TSSK2* | Testis specific serine kinase 2 | 1.688 |
| *TTC9* | Tetratricopeptide repeat domain 9 | 1.423 |
| *TTR* | Transthyretin | 4.029 |
| *UBE2L6* | Ubiquitin conjugating enzyme E2 L6 | -1.555 |
| *USP20* | Ubiquitin specific peptidase 20 | -1.336 |
| *USP40* | Ubiquitin specific peptidase 40 | 1.432 |
| *VCAM1* | Vascular cell adhesion molecule 1 | -1.765 |
| *VNN2* | Vanin 2 | -2.472 |
| *VWA1* | Von Willebrand factor A domain containing 1 | -1.528 |
| *WNT5A* | Wnt family member 5A | 2.023 |
| *YOD1* | YOD1 deubiquitinase | 1.745 |
| *ZC3HAV1* | Zinc finger CCCH-type containing, antiviral 1 | 1.49 |
| *ZCCHC17* | Zinc finger CCHC-type containing 17 | -1.27 |
| *ZCWPW1* | Zinc finger CW-type and PWWP domain containing 1 | -1.806 |
| *ZNF385A* | Zinc finger protein 385A | 1.393 |

^1^ Fold changes are up or down in compensating animals compared to restricted animals
