# Supplementary material for: Effect of dietary restriction and subsequent re-alimentation on the transcriptional profile of bovine ruminal epithelium
Source: PLoS One. 2017 May 17;12(5):e0177852. doi: 10.1371/journal.pone.0177852 (PMC5435337; doi:10.1371/journal.pone.0177852)
Supplement: S3 Table — (DOCX) [file pone.0177852.s003.docx]

**S3 Table.** Networks generated from gene expression data of restricted versus *ad libitum* fed bulls by IPA

| Network ID | Top Functions | Molecules in Network | Score | Focus Molecules |
| --- | --- | --- | --- | --- |
| 1 | Nervous System Development and Function, Cell-To-Cell Signaling and Interaction, Inflammatory Response | *ALOX15B, chemokine, CIRBP, Collagen Alpha1, CRYAB, DHX58, DUSP6, ELF3, ERK1/2, FCER1A, FERMT2, HSP, HSPA4L, HSPB8, HSPH1, Ifn, IFN Beta, Ige, IgG1, IL1, IL12 (complex), IL12 (family), IL17A, LBP, LGALS4, LRMP, MRC2, MTUS1, Pro-inflammatory Cytokine, SOCS2, Sos, STAT5a/b, Tlr, Tnf (family), ZC3H12A* | 42 | 19 |
| 2 | Post-Translational Modification, Cell Morphology, Tissue Morphology | *ATP6AP2, BCCIP, BEX2, BFAR, CCDC8, CHMP3, CHPT1, DHX32, ECI2, EMR2, GNAO1, GPR56, GPR143, HIST1H2BD, HIST1H2BN, MAP7D1, MC1R, MLANA, MTNR1B, N4BP3, NAT9, PANK3, PGS1, PLEKHA7, RAB20, SNRNP25, SUCLG2, THOC3, TXNDC5, UBC, USP1, USP20, USP45, USP53, YOD1* | 31 | 15 |
| 3 | Cardiovascular System Development and Function, Cell Death and Survival, Reproductive System Development and Function | *AGPAT9, Akt, ANGPTL1, BMPR1B, caspase, CD3, CDH2, Cg, Collagen type I, Creb, DSG1, ERK, FSH, Histone h3, INPP4B, Insulin, Jnk, Mapk, NFkB (complex), NR4A1, NTRK2, P38 MAPK, PDGF BB, PI3K (complex), PIK3C2G, Pka, Pkc(s), PRDX6, RBM3, SATB1, SCUBE1, TCR, Tgf beta, TGM2, Vegf* | 28 | 14 |
| 4 | Carbohydrate Metabolism, Molecular Transport, Dermatological Diseases and Conditions | *ALOX5AP, AQP9, C4orf19, CD302, CDH13, CLEC4E, CLTCL1, COTL1, DMD, DSC2, FLNA, FMO1, FOXJ3, G0S2, GAN, GMDS, HERC3, HLA Class I, HNF1A, HNF4A, IFNB1, KCNE4, MLANA, ORM2, PAMR1, PGM5, PKP2, PPP1R3C, SFN, SHROOM1, STARD10, Tgtp1/Tgtp2, TNF, Ubiquitin, WNT2B* | 21 | 11 |
| 5 | Cellular Assembly and Organization, Cellular Function and Maintenance, Cellular Movement | CCSER1, NDEL1 | 2 | 1 |
