# Supplementary material for: Effect of dietary restriction and subsequent re-alimentation on the transcriptional profile of bovine ruminal epithelium
Source: PLoS One. 2017 May 17;12(5):e0177852. doi: 10.1371/journal.pone.0177852 (PMC5435337; doi:10.1371/journal.pone.0177852)
Supplement: S4 Table — (DOCX) [file pone.0177852.s004.docx]

**S4 Table.** Networks generated from gene expression data of compensating versus restricted fed bulls by IPA

| Network ID | Top functions | Molecules in network | Score | Focus molecules |
| --- | --- | --- | --- | --- |
| 1 | Cancer, Respiratory Disease, Organismal Injury and Abnormalities | *26s Proteasome, BAG3, BDH2, calpain, CCT2, CEP97, CHORDC1, CTTNBP2, FAM107B, FBXO17, Focal adhesion kinase, GSPT1, HMGB2, Hsp70, Hsp90, HSP90AA1, HSP90AB1, HSPA8, KIF21A, LIMCH1, MDN1, Mek, NRG1, PRCC, Rnr, RPS3A, SGK223, SGSH, SLC25A15, Sos, SRPK2, TFB2M, TGM2, TSSK2, TSSK1B* | 42 | 27 |
| 2 | Cellular Development, Skeletal and Muscular System Development and Function, Cell Morphology | *AKR1C3, ALDH1A2, Alpha tubulin, ASPN, BMP2, BMPR1B, Calcineurin A, Calcineurin protein(s), CKM, CNN3, DHRS9, Dynein, EPS8, ERK, FGFR2, FKBP4, GHR, GLIPR1, Id, MAL, Nfat (family), NPC2, NR3C2, NTRK2, PPID, PPP3CC, Rab11, RCAN2, RDH, Smad1/5/8, SNX2, SPRED1, STYX, TICAM2, TRIP10* | 37 | 25 |
| 3 | Cardiovascular System Development and Function, Tissue Development, Cellular Movement | *AHSA1, ANPEP, ANTXR1, CD63, COL12A1, COL4A1, COL4A2, collagen, Collagen type II, Collagen type III, Collagen type IV, Collagen type V, CTSB, CYBA, DOCK3, ERK1/2, Laminin, Laminin1, LOX, MRC2, MTSS1, MTUS1, NID1, PCOLCE2, PDGF (family), PDGF-CC, PDGFA, PDGFC, POSTN, PROCR, RELN, SBSN, SERPINH1, SMAD1/5, THBS4* | 35 | 24 |
| 4 | Drug Metabolism, Cell-To-Cell Signaling and Interaction, Cellular Movement | *ACPP, Alpha catenin, ARNTL, BCL6, CCL20, CH25H, Cmah, CXCL2, CXCR4, DYRK3, EFS, FDFT1, FSH, GOT, GST, GSTM1, GSTM4, Ifn, IgG1, Igg3, Immunoglobulin, Lh, LRMP, LYZ, Mapk, MOG, P4HA1, P4HA2, RBP2, RORC, ST3GAL4, STAT5a/b, STIP1, Sult1a1, THBS2* | 35 | 24 |
| 5 | Post-Translational Modification, Hereditary Disorder, Neurological Disease | *AMPD2, AMPK, ANGPT2, BAG2, CCNF, CRYAB, DNAJB4, DNAJC21, DUOX1, GPSM2, HSP, Hsp22/Hsp40/Hsp90, HSPA4L, HSPB1, HSPB8, HSPE1, HSPH1, KCNMA1, KCNMB1, KIF20A, KIF4A, MHC Class II (complex), Mucin, NADPH oxidase, PLXDC2, PRC1, PRDX6, SAT1, SCIN, Secretase gamma, Tlr, trypsin, TSH, TTLL12, Vegf* | 35 | 24 |
| 6 | Carbohydrate Metabolism, Small Molecule Biochemistry, Cellular Assembly and Organization | *CACYBP, CIRBP, Cyclin D, Cyclin E, E2f, EDN3, GAS2L1, GINS2, Growth hormone, HABP4, Hat, Hdac, HIST1H4J, HIST2H4A, HISTONE, histone deacetylase, Histone h4, HLCS, HPSE, HYPK, ITGA8, KAT2A, KCNC4, KRT14, MAOB, N-cor, NAA50, PER1, Pkc(s), QPCT, Rar, Rb, SATB1, SLC6A9, TCF* | 29 | 21 |
| 7 | Cellular Function and Maintenance, Cellular Growth and Proliferation, Hematological System Development and Function | *Alp, CACNA1G, CD3, Cg, Ck2, CLPB, Creb, DHRS12, ELL2, F Actin, FOXN1, Gsk3, HERC3, HIST1H2AC, Histone h3, HMCN1, HMGN1, ID2, Insulin, KPTN, MMP2, p85 (pik3r), PAFAH1B3, PAM, PAPOLA, PDGF BB, PRSS35, RB1, RNA polymerase II, RNASEH2A, RUNX2, SPATS2L, Troponin t, Ubiquitin, WNT5A* | 29 | 21 |
| 8 | Lipid Metabolism, Small Molecule Biochemistry, Molecular Transport | *A2M, ADAMTS4, AGPAT9, APOE, BMX, C8, C12orf45, CD59, chymotrypsin, DGAT2, Fc gamma receptor, Fcer1, FCER1A, GPC3, HDL, HDL-cholesterol, IL-1R, Jnk, LDL-cholesterol, LPL, LRP, NfkB1-RelA, Nos, PLC gamma, PLCG2, PLVAP, PRKD1, RNASEL, ROR2, SFRP2, TTR, VAV, Vla-4, VLDL, VLDL-cholesterol* | 23 | 18 |
| 9 | Neurological Disease, Hereditary Disorder, Metabolic Disease | *ACADVL, Actin, ADRB, ALDH2, ALOX15B, Ampa Receptor, ATPase, CaMKII, caspase, Cofilin, COX10, Cytochrome bc1, cytochrome C, cytochrome-c oxidase, DSG1, HSPD1, ICA1, Ifn gamma, ISL1, Mitochondrial complex 1, Mlc, MT-ND5, MT-ND6, NDUFA4L2, NDUFAB1, OCLN, P38 MAPK, PCDH7, PP2A, Proinsulin, PSD, Rock, SYNGAP1, TIAL1, UQCR10* | 23 | 18 |
| 10 | Cell-To-Cell Signaling and Interaction, Cellular Compromise, Tissue Development | *ADCY5, Akt, AKT1S1, ANGPT4, ANGPTL1, CDH2, Collagen Alpha1, Collagen type I, DNA-methyltransferase, EHHADH, FERMT2, Fgf, Fgfr, Fibrin, Filamin, GLB1, growth factor receptor, Integrin, Integrin alpha 3 beta 1, Integrin alpha 4 beta 1, JINK1/2, Lfa-1, MIA, MMR, N-Cadherin, NDRG2, NQO2, PDK4, Ptk, SLC22A17, SLC35B2, SLC4A7, SPAG5, TGFBI, Wnt* | 21 | 17 |
| 11 | Dermatological Diseases and Conditions, Developmental Disorder, Organismal Injury and Abnormalities | *Angiotensin II receptor type 1, AP4B1, BEX2, BMP, CHI3L1, Collagen type X, DLX3, EDAR, elastase, ELF3, ENG, ETS, FTL, GFPT2, HPGD, IGFBP5, MAST2, MMP13, NCOR-LXR-Oxysterol-RXR-9 cis RA, NFkB (complex), Nuclear factor 1, Par, PRKAC, Rxr, secreted MMP, Serine Protease, SFTPC, Smad2/3, Smad2/3-Smad4, STAT1/3/5 dimer, Stat3-Stat3, THOP1, TMEM119, VitaminD3-VDR-RXR, ZNF385A* | 21 | 17 |
| 12 | Lipid Metabolism, Molecular Transport, Small Molecule Biochemistry | *APOH, AS3MT, BPHL, C4orf19, CD302, CDHR2, EMG1, ETNPPL, FAM107B, FBP2, FOXJ3, GLA, GMDS, HIST1H2BD, HNF1A, HNF4A, KIF20A, MID1IP1, MT1H, NOP16, PAMR1, PCNP, POC1A, PZP, R3HDM1, RTP3, SGK2, SLC37A4, Slco1a1, Slco1a4, SQRDL, SUGCT, TM7SF2, YPEL3, ZNF443* | 21 | 17 |
| 13 | Metabolic Disease, Amino Acid Metabolism, Molecular Transport | *BCR (complex), BTC, CAPG, CCL19, Cdc2, CHP2, creatine kinase, Cyclin A, Eif4g, EIF4G2, EIF4G3, ENaC, Erm, FAM3B, GCLM, Gm-csf, Histone H1, Hsp27, IL23, Ldh (complex), MAP2K1/2, MARCKS, Notch, PARP, PI3K (complex), PI3K (family), PIK3C2G, RBM3, RNASE1, Rsk, SKIL, SLC1A5, SLC9A1, Sod, ZC3HAV1* | 20 | 16 |
| 14 | Cellular Assembly and Organization, Cellular Function and Maintenance, Cell Cycle | *ACAD9, BCCIP, CASP14, CCDC8, DUT, FAM120A, FARP1, FOXRED1, HAUS3, HAUS4, HAUS5, HAUS7, HIST1H2BN, KBTBD6, KRT78, LTA4H, METTL23, OSBPL3, OSBPL9, OSBPL10, OSBPL11, PLCD3, PLCH1, PMPCB, SEP15, SKA3, SPC24, SYT3, SYT5, TANC1, THOC3, UBC, VCPKMT, YIF1A, YOD1* | 20 | 16 |
| 15 | Developmental Disorder, Hereditary Disorder, Metabolic Disease | *ANTXR1, CHI3L2, CHPT1, DIDO1, EPHX2, ETFA, ETFDH, FADS3, FAT4, GK5, GOLM1, Gyk, HIST1H2BO, KIFAP3, MYO18B, NIPSNAP3A, PARP2, PARP9, PARP12, PARP14, RAB20, RMND5B, SDK2, SH2D4A, SH3BGRL, SIPA1L1, TDP2, TMEM131, TNKS2, TULP3, UBC, USP25, XAF1, XRCC3, ZIC2* | 20 | 16 |
| 16 | Hematological Disease, Gastrointestinal Disease, Hereditary Disorder | *APP, CDC42EP4, CIRBP, CLEC3B, COMTD1, CRIP2, GPKOW, IRGM, KCNA4, KHDRBS3, KISS1, LNX1, LRRC1, LRRC3B, MND1, MTHFR, ORMDL3, OSTF1, PAXIP1, PKP4, PLBD1, PQBP1, RASL11B, RGS1, RPS27A, SCCPDH, SH3PXD2A, SKA3, SKAP2, SPATA7, TCEAL8, TCN2, TMEM144, WAC, ZCCHC17* | 20 | 16 |
| 17 | Post-Translational Modification, Cell-To-Cell Signaling and Interaction, Developmental Disorder | *ACOT9, ACSF3, APMAP, ATP13A1, C18orf32, CYSTM1, EMR2, FOXP4, GABARAPL1, GLP1R, GPR64, GPR119, GPRC5B, INIP, INTS3, MFSD5, NABP1, PIK3R5, PTPRS, RASA4, REEP2, RPL36A, RRP1B, SLC15A4, SNRNP25, SYNGR3, TBC1D2, TMEM147, UBC, USP20, USP30, USP38, USP40, USP45, USP9Y* | 20 | 16 |
| 18 | Cellular Movement, Cell-To-Cell Signaling and Interaction, Hematological System Development and Function | *ALOX15B, AR, C5AR2, CCL27, CYP1A1, DDT, FUT2, GBP1, GMFG, hemoglobin, Icam, IFIT1B, IRF3, ITIH5, LIX1, LMOD1, MAPK1, MIF, MMP28, MTUS1, OVGP1, PI3Kγ, PLA2G4F, PSPC1, RGS1, SCARA5, SLC35B2, SLC46A3, SPOCK1, SPON2, TDRD7, TLR9, TMEM158, TNF, TPST1* | 20 | 16 |
| 19 | Developmental Disorder, Hereditary Disorder, Metabolic Disease | *ADIRF, AIFM1, AMDHD2, ATRX, BRD4, C19orf54, CERS2, CIAO1, CLDN7, DPP9, DPY19L1, ELOVL4, FGD2, GPN1, KDM5B, KIAA0922, MMACHC, MTR, NIF3L1, PSAP, PTPN12, RENBP, RNASET2, RPL12, RPL10A, RPS6KA6, SLC25A26, SLC30A5, SLC30A6, SMAGP, SND1, THAP2, UBC, VANGL1, ZBED1* | 18 | 15 |
| 20 | Cardiovascular System Development and Function, Organismal Development, Embryonic Development | *AKT1, BMX, C1GALT1C1, CCL19, CHI3L1, CLDN23, FKHR, GALNT3, GALNT6, GALNT8, GALNT16, HIF3A, HTATIP2, IKBKG, Integrin alpha 2 beta 1, KDR, LYRM1, ME2, Mt2, MTAP, MYLIP, NDRG2, NQO2, P2RY2, PDGFC, PDGFD, polypeptide N-acetylgalactosaminyltransferase, PRKD3, RELB, ROBO4, SERTAD4, STAT, UNC5B, Vegf Receptor, VEGFA* | 18 | 15 |
| 21 | Dermatological Diseases and Conditions, Hereditary Disorder, Amino Acid Metabolism | *14-3-3, Ap1, BLVRA, CCNB2, DHX58, ENPEP, EPB41L3, Fibrinogen, IFN Beta, IFN type 1, Ifnar, Iga, Ige, IgG, IgG2a, Igm, IL1, IL12 (complex), IL12 (family), Interferon alpha, KRT75, LDL, LGALS3, MMP28, MX2, p70 S6k, PIBF1, Pka, SAA, SIK2, Smad, SOCS2, STAB1, Tgf beta, UBE2L6* | 16 | 14 |
| 22 | Embryonic Development, Cell Morphology, Reproductive System Development and Function | *AASS, ABI3BP, ACOT7, AIG1, ATP5G1, BDKRB1, CCR10, COL16A1, CTHRC1, DGKZ, DLG4, FAM195A, FOS, FZD5, FZD-LRP1/5/6, GLRX2, GPR64, HTT, ISLR, LBH, LPAR4, LYPD6, MCOLN1, NFATC1, P2RY1, P2RY6, PARD6A, PIK3CD, POLR2M, PRSS22, RAC1, RASD2, SRGAP1, TGFB1, WNT1* | 16 | 14 |
| 23 | Cellular Movement, Immune Cell Trafficking, Hematological System Development and Function | *ACKR4, ALOX15B, BDKRB1, Beta Arrestin, C5AR2, CCL19, CCL23, CCL25, CCL27, CCL28, CCL4L1/CCL4L2, CCR10, CD300LG, chemokine, Clathrin, CXCL17, EGFR ligand, FFAR2, FFAR3, Glycam1, GPR4, GPR34, GPR119, IKK (complex), ITGB2, Metalloprotease, NKX2-3, P2RY2, P2RY6, Pro-inflammatory Cytokine, SRC (family), TCR, Tnf (family), VCAM1, VNN2* | 11 | 11 |
| 24 | Cellular Movement, Hematological System Development and Function, Humoral Immune Response | *ADCY, Calmodulin, CD3G, Collagen(s), CXCL12, EFEMP1, estrogen receptor, G protein, G protein alpha, G protein alphai, G protein beta gamma, G-protein beta, GABBR1, Girk, GNAL, Gpcr, GPR143, Mmp, NMDA Receptor, P110, P2RY2, PALM, Pdgf (complex), Pdgfr, PLC, Rac, Rap1, Ras, Ras homolog, S1PR1, Sapk, Sfk, Shc, SPA17, tubulin (complex)* | 10 | 10 |
| 25 | Metabolic Disease, Hereditary Disorder, Nutritional Disease | *AKAP13, ANKS1A, APTX, CA1, CA2, CA3, CA4, CA13, CA5B, Carbonic anhydrase, CD47, CPSF3, EPB41L3, EPO, ERRFI1, GLUL, Hbb-b2, Importin alpha, KRT17, MDM4, NELFB, NR2E3, NR3C1, PDE4B, PDE6C, RXRB, SAMSN1, SFN, SHROOM1, SON, TNFRSF11B, TNS4, TSPYL2, UGCG, WDR37* | 6 | 7 |
